# Prevalence, evolution, replication and transmission of H3N8 avian influenza viruses isolated from migratory birds in eastern China from 2017 to 2021

**DOI:** 10.1080/22221751.2023.2184178

**Published:** 2023-03-13

**Authors:** Yanwen Wang, Mengjing Wang, Hong Zhang, Conghui Zhao, Yaping Zhang, Jinyan Shen, Xiaohong Sun, Hongke Xu, Yujiao Xie, Xinxin Gao, Pengfei Cui, Dong Chu, Yubao Li, Wenqiang Liu, Peng Peng, Guohua Deng, Jing Guo, Xuyong Li

**Affiliations:** a College of Agronomy, Liaocheng University, Liaocheng, People’s Republic of China; b Harbin Veterinary Research Institute of Chinese Academy of Agricultural Sciences, State Key Laboratory of Veterinary Biotechnology, National Poultry Laboratory Animal Resource Center, Harbin, People’s Republic of China; c Biological Disaster Control and Prevention Center, National Forestry and Grassland Administration, Shenyang, People’s Republic of China

**Keywords:** Avian influenza virus, H3N8, evolution, replication, transmission

## Abstract

The continued evolution and emergence of novel influenza viruses in wild and domestic animals poses an increasing public health risk. Two human cases of H3N8 avian influenza virus infection in China in 2022 have caused public concern regarding the risk of transmission between birds and humans. However, the prevalence of H3N8 avian influenza viruses in their natural reservoirs and their biological characteristics are largely unknown. To elucidate the potential threat of H3N8 viruses, we analyzed five years of surveillance data obtained from an important wetland region in eastern China and evaluated the evolutionary and biological characteristics of 21 H3N8 viruses isolated from 15,899 migratory bird samples between 2017 and 2021. Genetic and phylogenetic analyses showed that the H3N8 viruses circulating in migratory birds and ducks have evolved into different branches and have undergone complicated reassortment with viruses in waterfowl. The 21 viruses belonged to 12 genotypes, and some strains induced body weight loss and pneumonia in mice. All the tested H3N8 viruses preferentially bind to avian-type receptors, although they have acquired the ability to bind human-type receptors. Infection studies in ducks, chickens and pigeons demonstrated that the currently circulating H3N8 viruses in migratory birds have a high possibility of infecting domestic waterfowl and a low possibility of infecting chickens and pigeons. Our findings imply that circulating H3N8 viruses in migratory birds continue to evolve and pose a high infection risk in domestic ducks. These results further emphasize the importance of avian influenza surveillance at the wild bird and poultry interface.

## Introduction

Avian influenza viruses (AIVs) pose persistent threats to birds, mammals and humans due to their rapid mutation, complicated reassortment, and cross-species transmission from birds to mammals. In the past two decades, highly pathogenic H5N1, H5N6 and H5N8 viruses have continued to evolve in wild and domestic birds and caused more than eight hundred human infections [1–4]. The H7N9 virus that emerged in chickens in 2013 led to five waves of human infections between 2013 and 2017 and underwent rapid mutation [5–7]. H9N2, the predominant subtype among the low-pathogenic viruses, circulates widely in poultry and wild birds and has adapted to replicate in mammals, resulting in more than 90 human infection cases since 1996 [3,8,9]. In recent years, avian-origin H3N8, H7N4, H10N3, and H10N8 viruses have also been reported to infect humans who have close contact with domestic birds [10–13]. The ongoing threat from different subtypes of avian influenza viruses emphasizes the importance of surveillance in their natural reservoirs.

Wild birds, especially migratory waterfowl, such as wild ducks, gulls and shorebirds, are known to be natural hosts of avian influenza viruses. Wild bird migration between countries and breeding play key roles in the maintenance and dissemination of viruses [14,15]. The highly pathogenic H5N8 viruses that caused recent outbreaks in poultry in Europe and Asia were confirmed to be closely related to those in migratory birds, and strains in wild birds have been found to be closely related to novel H5N6 and H5N1 reassortants that have evolved and been causing outbreaks in America, Europe, Africa and Asia since 2021 [2,16–19]. Previous studies found that the H7 low-pathogenicity virus from wild birds contributed to the emergence of highly pathogenic viruses [20]. Our recent study found that H10 viruses of the North American lineage have been introduced into Asia by migratory birds [21]. Surveillance of dominant and emerging viruses in migratory birds and monitoring cross-transmission to commercial poultry and mammals will contribute to early detection of a pandemic threat posed by novel avian influenza viruses.

H3N8 influenza viruses have been detected in a wide range of mammalian hosts, including dogs [22], horses [23], pigs [24], donkeys [25], harbour seals [26], and recently humans [10,27], and have been associated with ongoing outbreaks in dogs and horses. H3N8 avian influenza viruses primarily circulate in wild birds and domestic ducks, and their genetic and biological characteristics are still relatively poorly understood. Previously, studies found that an H3N8 avian influenza virus isolated from harbour seals preferentially bound to human-type receptors, which could be transmitted among ferrets via respiratory droplets and replicated efficiently in human lung cells [26]. A recent study demonstrated that wild bird-origin H3N8 viruses acquired the ability to bind to human-type receptors, and some viruses were transmitted efficiently via contact among guinea pigs. The PB1 S524G mutation conferred avian H3N8 virus airborne transmissibility in ferrets [28]. Li et al. found that a wild bird H3N8 virus developed enhanced human-type receptor binding ability and exhibited good adaptation in mice [48]. Human infection with H3N8 AIV was first reported in Henan Province, China, on April 10, 2022. A 4-year-old boy developed fever and lethargy followed by severe acute respiratory distress syndrome, including dyspnea, hypoxemia and pneumonia, in a short period of time. Avian H3N8 virus was detected in alveolar lavage fluid and peripheral blood samples [10]. In May 2022, a second H3N8 infection case was reported in Hunan Province, China. A 5-year-old boy developed fever and chills on May 9, 2022, and recovered after symptomatic treatment [27]. Subsequent surveillance and phylogenetic studies indicated that the two human H3N8 isolates were genetically close to the emerged chicken H3N8 reassortants, which may have originated from duck H3Nx viruses (HA donor), wild bird HxN8 viruses (NA donor) and chicken H9N2 viruses (internal gene constellation donor) [29–31]. These findings, together with human infection reports, suggest that H3N8 AIV poses an increasing threat to public health.

Since 2017, we have conducted annual avian influenza surveillance in migratory birds in the Yellow River Delta wetland in eastern China to monitor the infection risk of wild bird-origin AIVs to domestic birds and mammals [1,21,32–34]. In this study, we analyzed five years of avian influenza virus surveillance data from the Yellow River Delta wetland and fully uncovered the ecological, genetic and biological characteristics of representative avian H3N8 viruses in migratory birds. These findings provide important information about the evolution and dissemination of wild bird-origin H3N8 viruses and provide insights for the surveillance of these viruses at the wild bird and commercial poultry interface.

## Results

### H3N8 viruses in migratory birds in eastern China

**Figure 1.**
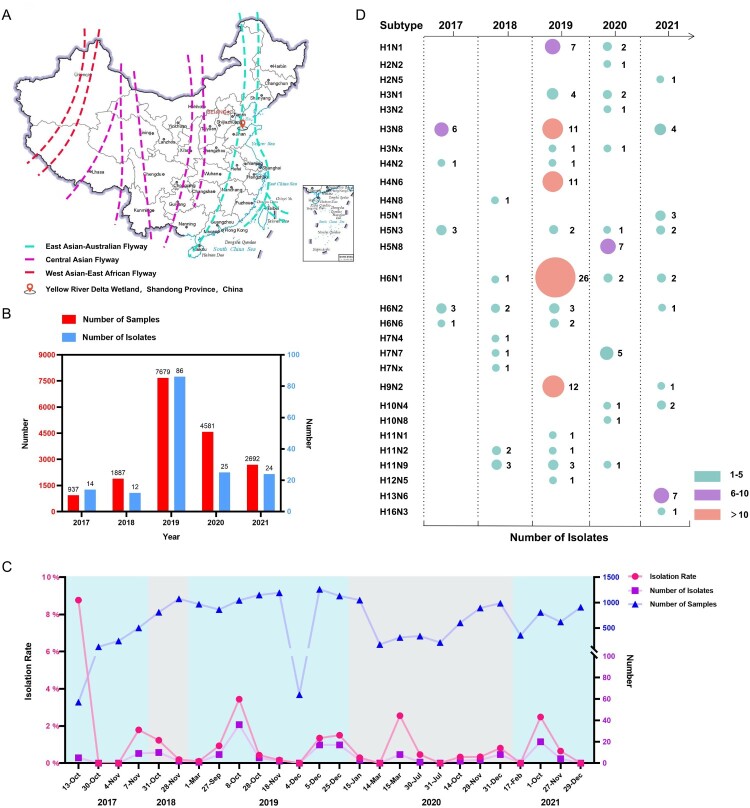
Prevalence of different subtypes of avian influenza viruses detected in wild birds in the Yellow River Delta wetland in eastern China, 2017–2021. (A) Sampling sites; (B) sampling size and number of isolates for each year; (C) sampling frequency, sampling numbers, AIV isolation numbers, and AIV isolation rates at each collection date; (D) subtypes identified.

From the autumn migratory season of 2017 to 2021, we conducted continuous active avian influenza surveillance in migratory birds in the Yellow River Delta wetland located in eastern China, which overlaps with the EAA migratory flyway (Figure 1A, Figure S1). A total of 162 viruses that belonged to 28 subtypes were identified and isolated from the 15,899 samples (Figure 1B–D). The AIV isolation rates in each migration season varied from 0.1% to 8.77%, while 2225 samples collected at seven time points between 2017 and 2021 were negative for AIVs (Figure 1C). Twenty-one of the 162 viruses were isolated from 3032 wild duck and gull samples from four independent sampling dates and identified as the H3N8 subtype (Figure 1D, Table S1) (the sequence data have been deposited in the GISAID EpiFlu Database under accession numbers EPI2245719-EPI2245772, EPI2245774, EPI2245776-EPI2245888). Six and eleven H3N8 viruses were isolated from wild ducks in 2017 and 2019, respectively, while four viruses were isolated from 806 migratory gull samples in 2021 (Table S1). No H3N8 virus was detected in 2018 or 2020. The annual sampling and virus information indicated that migratory birds provide a large gene pool, driving the prevalence and evolution of different influenza viruses. These identified subtypes further revealed that H3N8, H4N6, H6N1 and H9N2 viruses were the dominant viruses in migratory birds in eastern China.

### Prevalence of H3N8 viruses in birds

**Figure 2.**
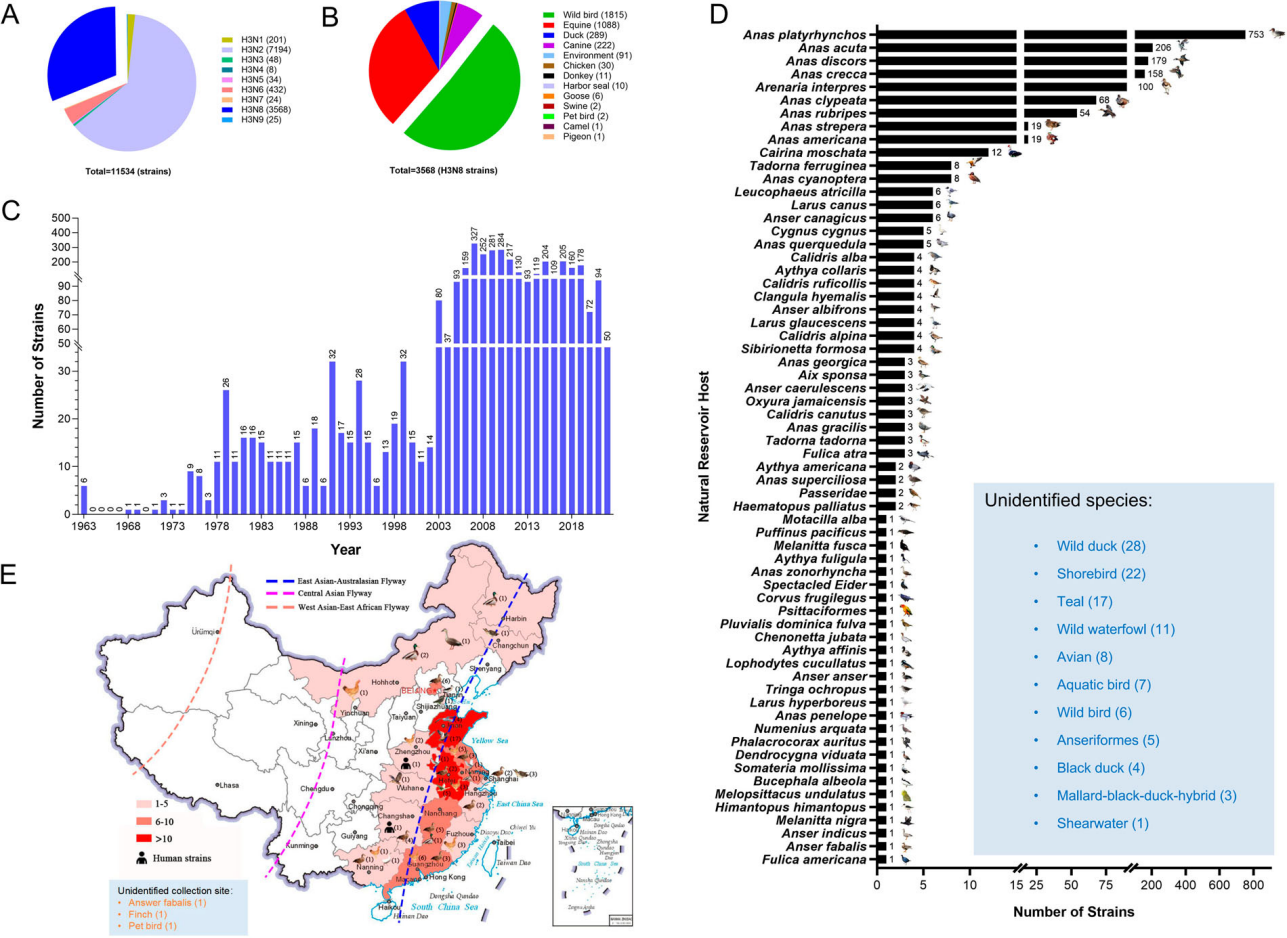
Global prevalence of H3N8 influenza A viruses. (A) HA and NA combinations of animal H3Nx strains in the database. (B) Summarized analysis of the animal hosts of H3N8 viruses. (C) Number of H3N8 viruses detected in wild birds from 1963 to 2022. (D) H3N8 viruses in migratory wild birds. The host species of the wild bird H3N8 viruses in the databases were classified and summarized according to their isolation information. Unidentified species indicate the H3N8 viral sequences in the databases without specific host information. (E) Distribution of H3N8 avian influenza viruses detected in China. All the public data in GenBank and GISAID used in this study were up to date as of November 25, 2022.

To better understand the ecology and epidemiology of H3N8 viruses, we mapped the globally circulated H3N8 viruses in different hosts. To date, nine H3 and NA surface combinations, including H3N1, H3N2, H3N3, H3N4, H3N5, H3N6, H3N7, H3N8 and H3N9, have been identified in animals, and a total of 11,534 HA sequences of H3Nx (N1–N9) viruses obtained from birds and mammals are available in databases (GenBank and GISAID, updated to November 25, 2022) (Figure 2A). Among these nine identified subtypes, H3N2 (7194 HA sequences) and H3N8 (3568 HA sequences) viruses are the predominant subtypes, and they have been detected in multiple animals. A total of 3568 H3N8 viral HA sequences were available in the databases, and these viruses were mainly detected in wild bird (1815 strains), equine (1088 strains), duck (289 strains), and canine (222 strains) viruses. Only 30 H3N8 strains were detected in chickens. Some H3N8 viruses were occasionally found in harbour seals, swine and camels (Figure 2B). The H3N8 virus was first detected in 1963 and has circulated in animals for more than half a century (Figure 2C). Because this study focused on H3N8 viruses circulating in wild birds, we summarized the identified specific hosts of global H3N8 viruses in wild birds. To date, H3N8 viruses have been detected in at least 64 different species of wild birds. Significantly, migratory wild ducks, including mallards (*Anas platyrhynchos*) (753 strains), pintails (*Anas acuta*) (206 strains), *Anas discors* (179 strains), *Anas crecca* (158 strains), *Anas clypeata* (68 strains) and *Anas rubripes* (54 strains), are the primary natural hosts of avian H3N8 viruses (Figure 2D). A total of 91 avian H3N8 viral HA sequences are available in the databases (including the sequences detected in this study), and these viruses were detected in wild and domestic birds in 17 provinces in China, including 55 strains obtained from wild ducks and domestic ducks and 21 viruses detected in chickens. Of note, most of the chicken viruses (16 of 21) were detected in 2022. Two human H3N8 strains were obtained in Henan and Hunan provinces in 2022. Interestingly, nearly all the H3N8 viruses were detected in the provinces of China located along the East Asia-Australasia (EAA) migratory flyway (Figure 2E). These categorized results suggest that H3N8 viruses have evolved to infect a wide range of animals and that avian-lineage viruses have been dominantly preserved in migratory wild ducks.

### Genetic diversity and evolution of H3N8 viruses

**Figure 3.**
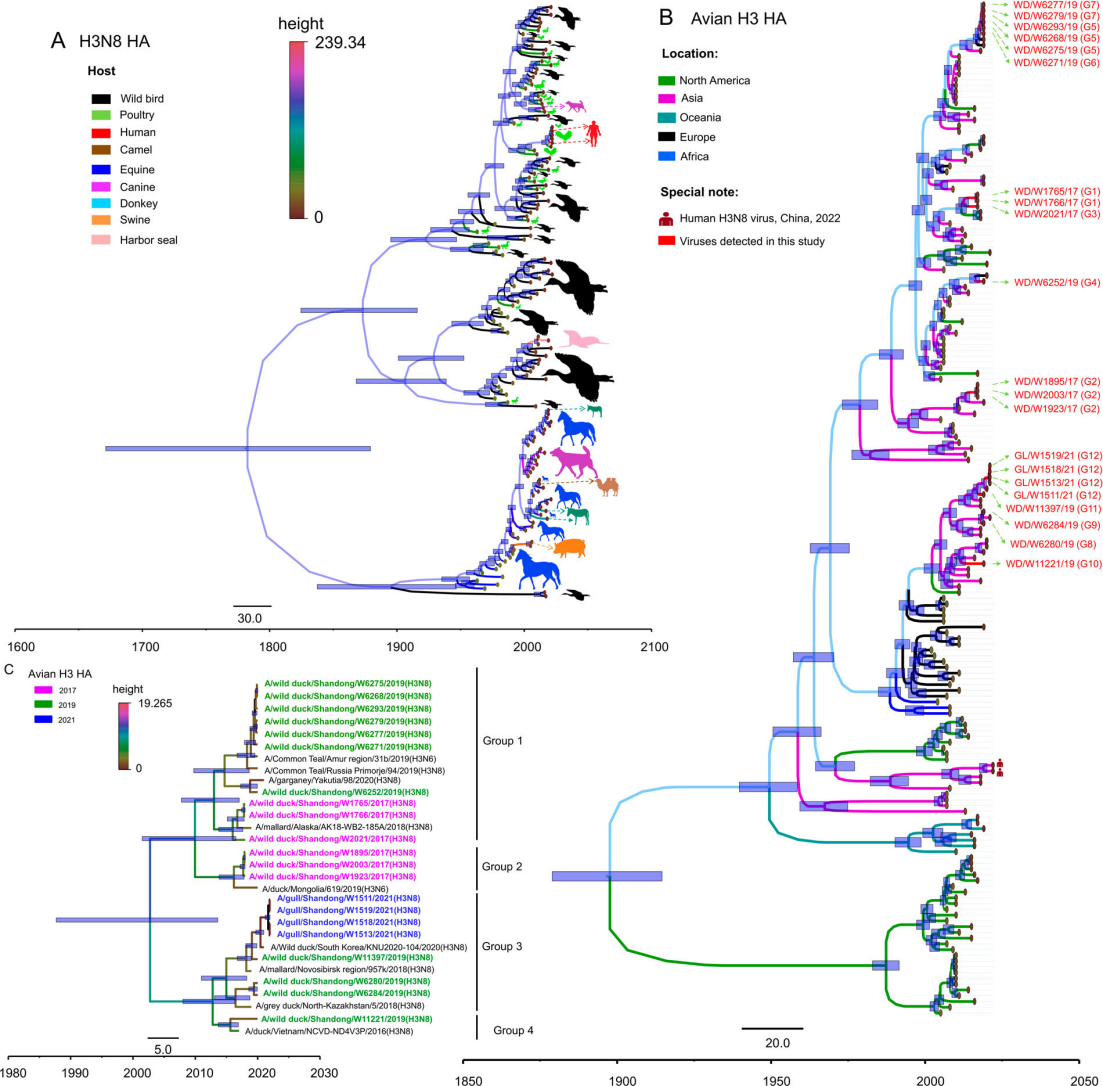
HA phylogenies of H3N8 viruses in all hosts (A), avian hosts (B) or wild bird hosts (C). Data for the time-scaled MCC tree inferred for the HA segment of the viruses in panel A were collected from all H3N8 hosts for which sequence data were available in GenBank and GISAID. The colour of each branch indicates the host, while the colour of each tip is the posterior time for that node. The colour of each branch in panel B indicates the location of the available avian H3Nx viruses. H3N8 viruses sequenced in this study are shown in red.

H3N8 viruses have circulated globally for more than half a century and have a wide range of reservoir hosts. We first downloaded the HA sequences of H3N8 viruses detected from wild birds, poultry, mammals and humans and selected 153 representative HA sequences to construct a Bayesian time-resolved tree to reveal the genetic diversity of HA genes of the global H3N8 influenza viruses in different hosts. The HA gene of H3N8 viruses has evolved into different lineages, and the viruses mainly detected in wild birds and poultry formed at least three major branches (Figure 3A). To better understand the genesis and evolution of the 21 H3N8 viruses identified in this study, we further constructed a Bayesian time-resolved tree of the HA gene plus 196 representative avian H3 viruses detected in North America, Asia, Oceania, Europe, and Africa. The phylogeny indicates that the HA genes of avian H3 viruses have undergone complex evolution and form several branches according to the location of virus isolation. Importantly, some North American strains clustered into the Eurasian lineage, suggesting that Eurasian H3 bird viruses have been introduced into North America. All 21 H3N8 viruses detected in this study clustered in the Eurasian lineage and formed at least four major branches (Figure 3B). The HA genes of the 21 viruses shared 91.1%–100% nucleotide similarity and were divided into four groups according to genetic identity (the nucleotide identity of the sequences between each group was lower than 95.4%) (Figure 3C). Notably, the HA genes of the two H3N8 viruses that caused human infection in China shared 85.2–88.1% identity with the wild bird viruses in this study and formed a branch with the chicken and duck H3 viruses isolated in China (Figure 3A and B).

**Figure 4.**
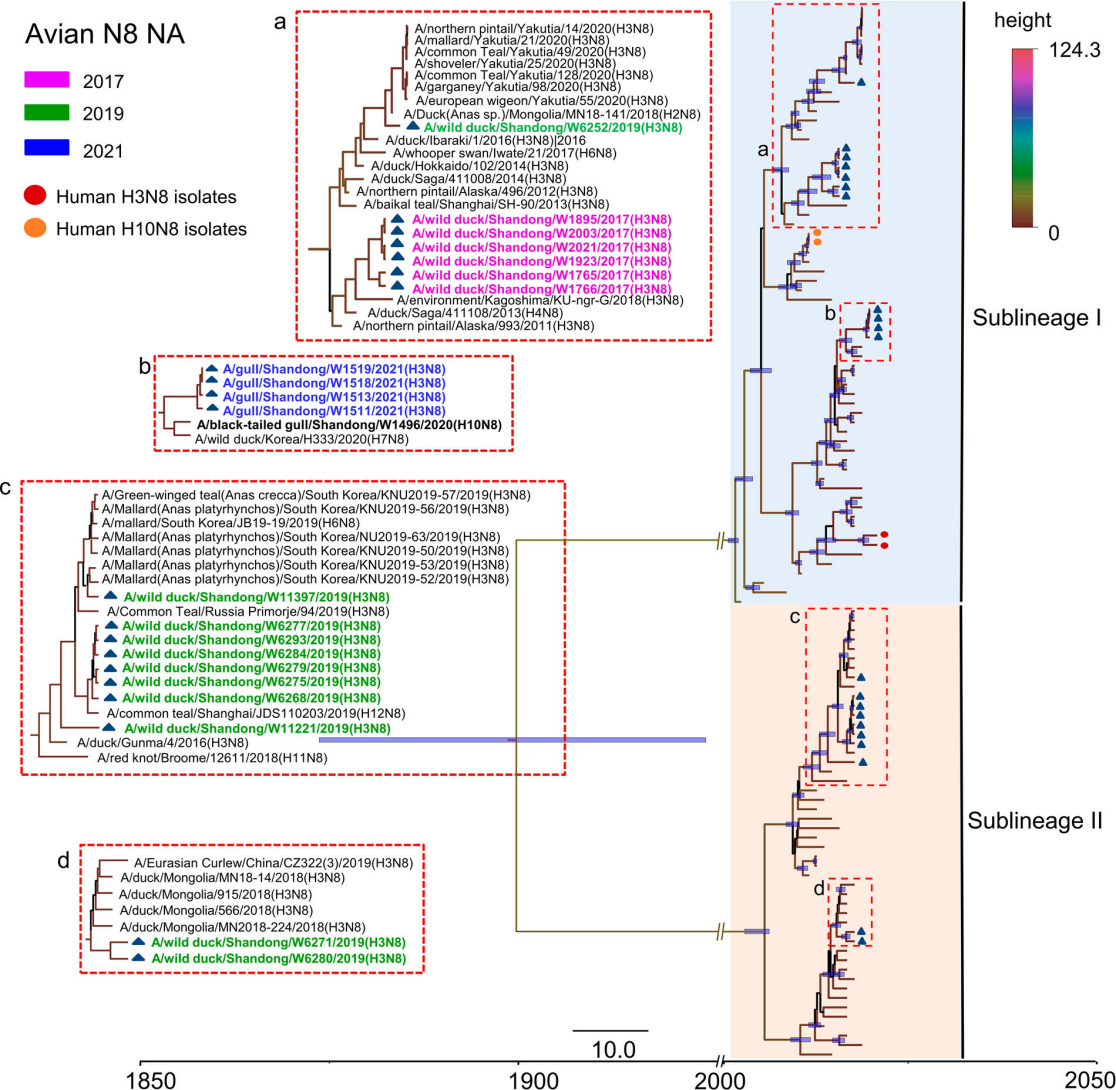
Phylogenetic analysis of the NA gene of the H3N8 virus. The sequences in purple, green, and blue represent the H3N8 viruses detected in this study.

The NA genes of the 21 H3N8 viruses shared 76.8–100% identity at the nucleotide level. The NA genes have evolved to form two distinct branches, as shown in the Bayesian time-resolved tree. The viruses isolated from gulls in 2021 shared high genetic similarity with a black-tailed gull H10N8 virus isolated in 2020 and clustered in a separate sublineage. The viruses isolated from wild ducks in 2017 and WD/W6252/2019 shared genetic similarity. The ten viruses isolated in 2019 that belonged to Sublineage II were divided into two different branches in the NA tree (Figure 4). Of note, the NA genes of the 21 H3N8 viruses were closely related to those of viruses detected in migratory birds and ducks in China, Mongolia, South Korea, Japan, Vietnam and Russia, located along the EAA migratory flyway (Figure 4). Six internal genes, i.e. PB2, PB1, PA, NP, M, and NS, of these 21 viruses shared 85.7–100%, 93.3–100%, 92.9–100%, 92.1–100%, 95.9–100%, and 70.6–100% identity, respectively. Notably, the internal genes of the viruses isolated in this study have undergone complicated reassortment with related viruses circulating in migratory birds and domestic ducks. The PB2 gene sequences of the 21 viruses clustered into five different branches in the phylogenetic tree, while the PB1, PA and NS gene sequences clustered into two separate branches each. The NP gene sequences of the 21 viruses clustered into three independent branches, and the M gene sequences formed one branch on the phylogenetic tree. Interestingly, the viruses we isolated from wild birds in the Yellow River Delta, including H7N4, H9N2, H10N4 and H10N8 viruses, shared high genetic similarity with the six internal genes of some H3N8 viruses detected in this study. The internal genes of the two human H3N8 isolates showed high homology with poultry and human H9N2 isolates and dissimilarity with the H3N8 viruses in this study (Figure S2).

**Figure 5.**
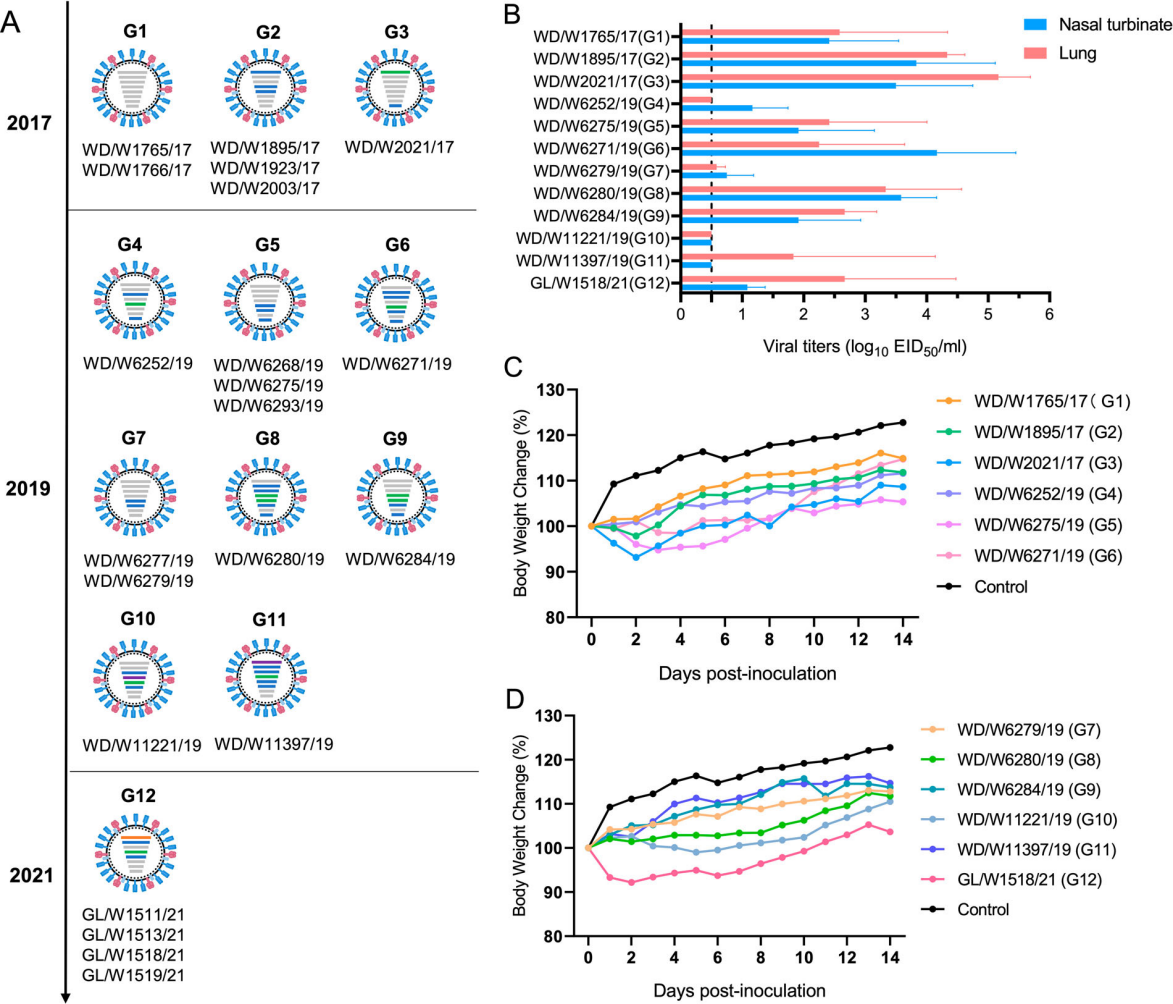
Genotypes of the H3N8 virus and replication of the representative viruses in mice. (A) Twelve genotypes of the 21 H3N8 viruses. (B) Replication of the representative H3N8 viruses in mice. The mice were inoculated with the representative viruses, and viral titers were detected in eggs. Data on the viral titers in the brain, spleen and kidney were negative and are not shown. The dashed line indicates the lower limit of detection. (C, D) Body weight change in the mice inoculated with the representative H3N8 viruses.

Phylogenetic analysis of each of the gene segments of the 21 viruses isolated in eastern China from 2017 to 2021 identified 12 genotypes (the distance between groups in each phylogenetic tree was as follows: PB2: 94%, PB1: 96.7%, PA: 94.5%, HA: 95.4%, NP: 95.7%, NA: 79.3%, NS: 72%). The six viruses isolated from wild ducks in 2017 were divided into three genotypes (G1–G3), while the 11 viruses isolated from wild ducks in 2019 were divided into eight genotypes. The four gull isolates detected in 2021 belonged to one genotype (Figure 5A). These results indicated that the H3N8 viruses that naturally circulate in migratory birds and ducks have evolved into different branches and have undergone complicated reassortment with viruses in waterfowl.

### Molecular characteristics of the H3N8 viruses

Several key molecular markers in each segment have been identified to play a key role in the receptor binding changes, replication, pathogenicity, and transmission of avian influenza viruses in birds and mammals. All 21 viruses shared the amino acid sequence PEKQTR/GLF at the cleavage site in the HA gene, suggesting that these viruses have low pathogenicity in chickens. All 21 viruses have acquired amino acid mutations (I155T and T160A (H3 numbering)) in the HA gene that have been identified to promote the binding of H5N1 and H9N2 viruses to human-type receptors [35]. Several amino acid substitutions that have been reported to increase replication, virulence or transmissibility in mammals were observed in these H3N8 isolates, including PB1-R207K, PB1-H436Y, NP-V41I, M1-N30D + T215A, and NS1-V149A [36–39]. Substitution of PA-515 T was detected in 20 of the 21 H3N8 viruses, excluding WD/W1766/17, which possesses PA-515S (Table S2). The mammalian host adaptive mutations PB2-E627K and D701N were not detected in any of the H3N8 isolates.

### Replication and virulence of representative H3N8 viruses in mice

Twelve representative viruses of each genotype (G1–G12) were selected for replication and virulence assessments in mice. The mice were inoculated with the viruses, and the organs, including the nasal turbinate, lung, spleen, kidney and brain, of the mice were collected at 3 dpi; viral titers were measured in eggs. Ten viruses, excluding WD/W11221/19 and WD/W11397/19, replicated in nasal turbinates in mice. Ten viruses, excluding WD/W11221/19 and WD/W6252/19, replicated in the lungs of mice. The viral titers in the nasal turbinate and lung ranged from 0.58 to 5.17 log_10_ EID_50_/ml (Figure 5B). No virus was detected in spleen, kidney or brain tissue (data not shown). Six of the twelve tested viruses induced body weight loss in infected mice (1%–7.8%) (Figure 5C and D). Pathological studies were performed on the lung samples of the mice. Most of the lung samples showed mild or moderate damage, including extensive inflammatory cell infiltration, necrosis and detachment of airway or alveolar epithelial cells, or widening of the alveolar diaphragm (Figure S4). These data imply that although some H3N8 isolates can replicate efficiently and cause body weight loss and lung inflammation in mice, the naturally isolated H3N8 viruses in migratory birds need to undergo further host adaptation before they develop increased virulence in mice.

### Receptor binding properties of representative H3N8 viruses

**Figure 6.**
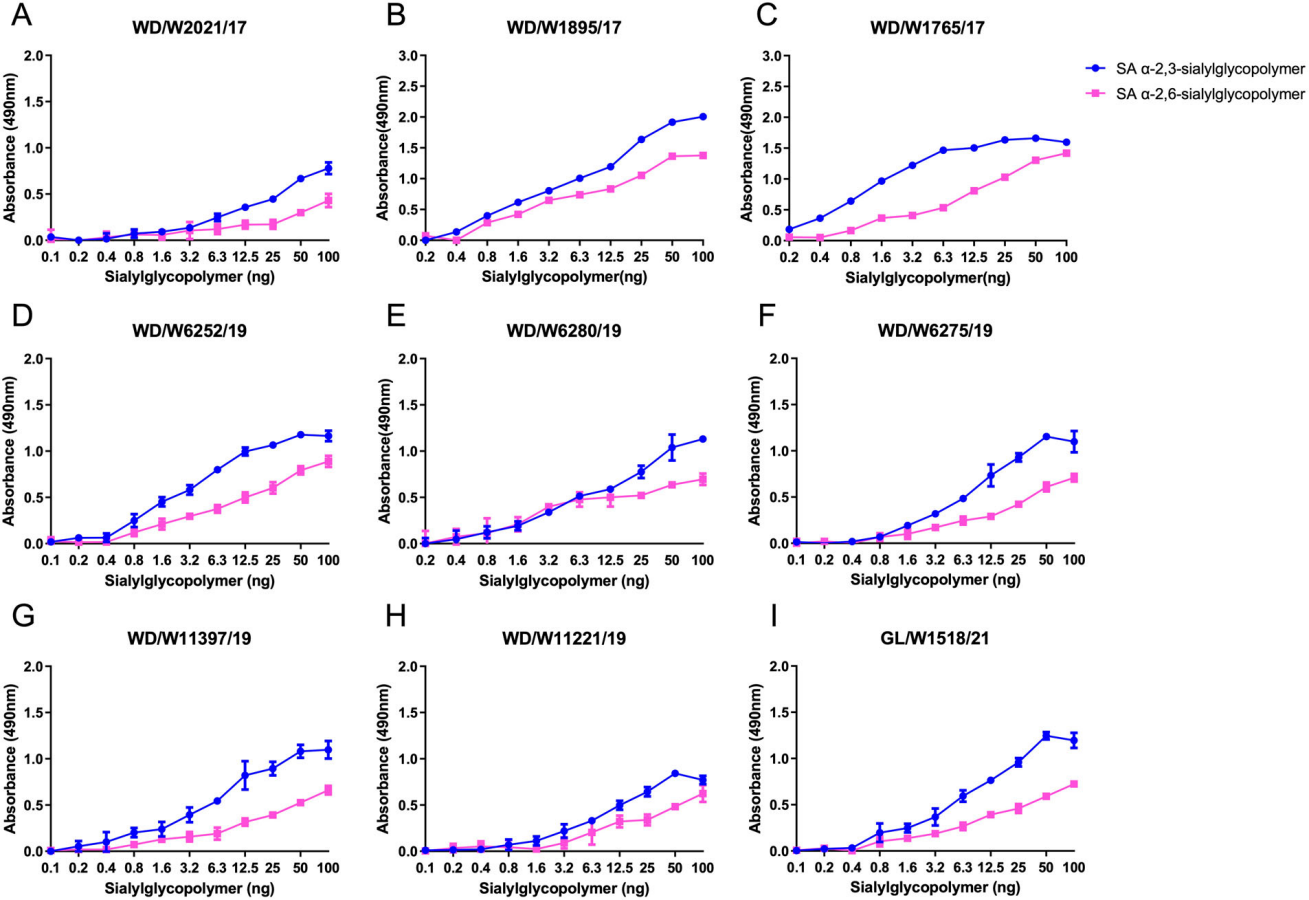
Receptor binding preferences of the representative wild bird H3N8 viruses. Two specific glycopolymers (α-2,3-siaylglycopolymer and α-2,6-siaylglycopolymer) were used to test the receptor binding properties of the representative H3N8 viruses. The data shown are the means of three replicates; the error bars indicate the standard deviation.

Receptor binding specificities of AIVs play key roles in the adsorption and invasion processes of viruses in target cells, and receptor-binding adaptation is a prerequisite for the cross-species transmission of avian viruses to mammals. Here, we selected nine representative viruses according to their evolutionary divergence in the HA phylogenetic tree. All the tested viruses primarily bind to avian-type receptors (SA α-2,3-sialylglycopoilmer), although they have acquired the ability to bind to human-type receptors (SA α-2,6-sialylglycopoilmer) (Figure 6). These results suggest that these naturally isolated H3N8 viruses from wild ducks and migratory gulls preferentially bind to avian-type receptors.

### Replication of representative H3N8 viruses in vitro

According to the results of the mouse infection and receptor binding studies, we selected three viruses, WD/W1895/17, WD/W6275/19 and GL/W1518/21, and evaluated their replication ability in avian and mammalian cells. Cells were inoculated with the representative viruses in 24-well plates, with 10^5^ EID_50_/ml of virus, and the supernatants of chicken embryo fibroblast (CEF), chicken embryo fibroblast (DEF), Madin-Darby canine kidney (MDCK), and human non-small cell lung cancer (A549) cells were collected and titrated in eggs. Interestingly, a significant difference in replication ability among the three viruses in avian and mammalian cells was observed. The titers of the representative viruses in MDCK cells were significantly higher than those in CEF, DEF and human A549 cells (Figure S3). These results indicate that the tested wild bird-origin H3N8 viruses have not adapted to replicate efficiently in chicken and duck embryo fibroblast cells and human cells.

### Representative wild bird H3N8 viruses replicated and were transmitted efficiently in ducks

**Figure 7.**
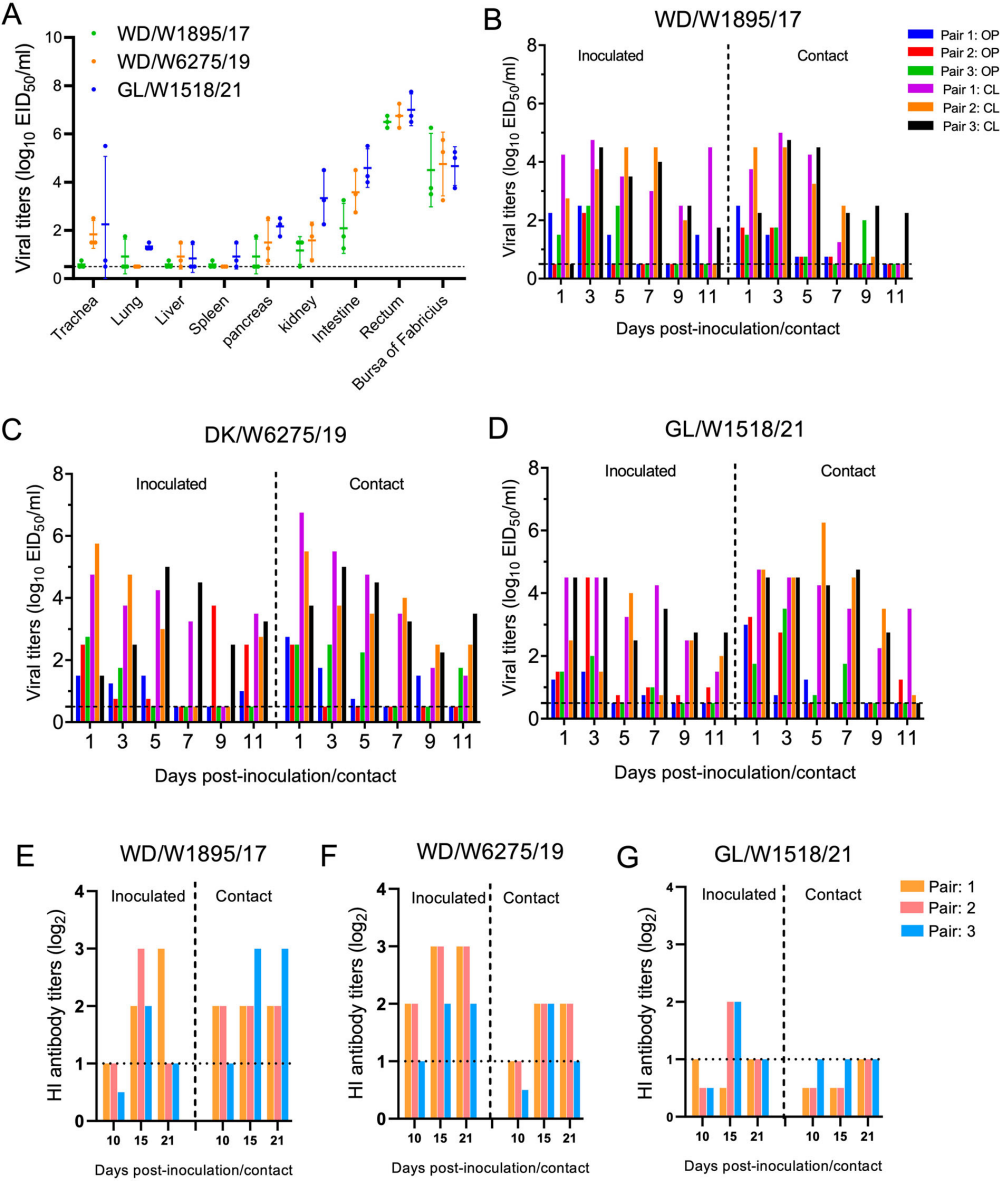
Replication and transmission of the representative H3N8 viruses in ducks. (A) Replication of the representative H3N8 viruses in ducks. SPF ducks were inoculated with the representative viruses; the organs were sampled at 3 dpi, and viruses were titrated in eggs. (B-D) Transmission of the representative H3N8 viruses in ducks. Oropharyngeal and cloacal swabs were collected from the ducks at the indicated time points, and the viruses were titrated in eggs. (E-F) Serum samples from inoculated and contact ducks were collected at 10, 15, and 21 dpi to detect HI antibodies. OP: oropharyngeal swab; CL: cloacal swab. The dashed lines indicate the lower limit of virus detection in panels A-D and the lower limit of HI antibody detection in panels E-G.

Ducks, including domestic ducks and wild ducks, are natural reservoirs of different AIVs. Here, we have summarized the host information of the avian H3N8 virus sequences in databases and found that more than 280 H3N8 viruses have been detected in domestic ducks (Figure 2B). However, the infection, replication and transmission abilities of H3N8 viruses originating from wild birds in domestic ducks are still unclear. In this study, three viruses, WD/W1895/17, WD/W6275/19 and GL/W1518/21, were used to investigate whether H3N8 viruses can replicate and be transmitted in domestic ducks. The viruses WD/W1895/17 and GL/W1518/21 were detected in all nine collected organs and tissues, and WD/W6275/19 was detected in all organs except for the lung and spleen. The viral titers of the three representative viruses in the intestine, rectum and bursa of Fabricius were significantly higher than those in the other organs, which suggests that the enteric canal, not the respiratory tract, of ducks is the major target organ for H3N8 viral infection (Figure 7A).

In the transmission study, all three representative viruses were detected in both oropharyngeal and cloacal swabs from the inoculated and contact ducks during the experimental period. Importantly, the virus shedding period in the inoculated and contact ducks for all three viruses was up to 11 days (Figure 7B–D). Interestingly, although the H3N8 viruses were able to replicate in the inoculated and contact ducks, they did not trigger the production of high hemagglutination inhibition (HI) antibody levels in the ducks to defend against and eliminate virus infection (Figure 7E–G). These findings suggest that domestic ducks are susceptible hosts of H3N8 viruses and that these wild bird H3N8 viruses can replicate in ducks and are transmitted efficiently via contact.

### Farmed chickens were not susceptible to representative wild bird H3N8 viruses

**Figure 8.**
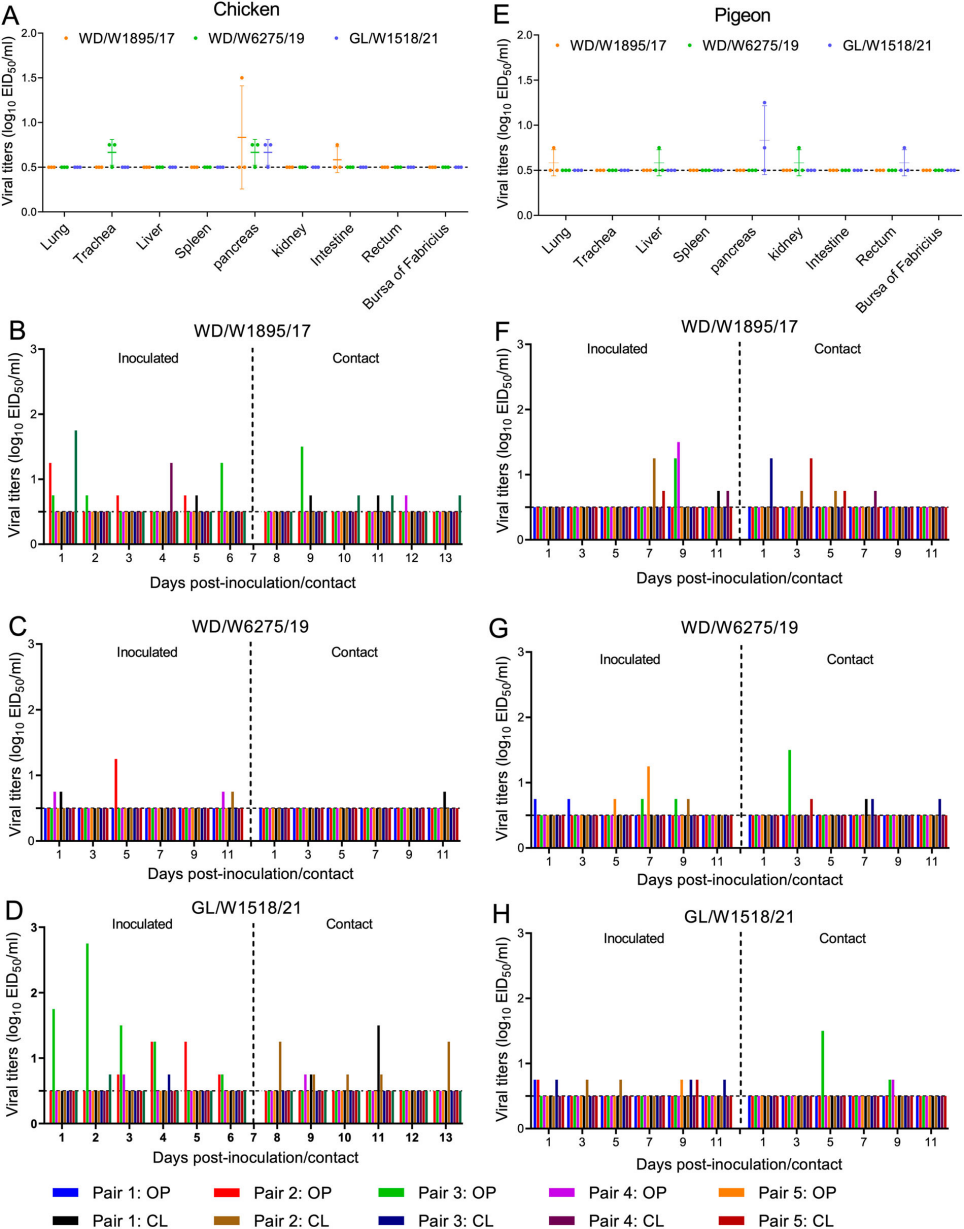
Replication and transmission of representative H3N8 viruses in chickens and pigeons. (A) Replication of the representative H3N8 viruses in chickens. Three commercial chickens were inoculated with the representative viruses, and viruses from the samples were titrated in eggs at 3 dpi. (B–D) Transmission of the representative H3N8 viruses in chickens. Oropharyngeal and cloacal swabs were collected from chickens at the indicated times, and viruses were titrated in eggs. (E) Replication of the representative H3N8 viruses in pigeons. Three pigeons were infected with the representative viruses; organ samples were collected, and the viruses were titrated in eggs at 3 dpi. (F-H) Transmission study of representative H3N8 viruses in pigeons. Swabs were collected, and the viruses were titrated in eggs. OP: oropharyngeal swab; CL: cloacal swab. The dashed line in each panel indicates the lower limit of detection.

Analysis of the sequences and samples of H3N8 viruses in GISAID and GenBank suggests that chickens may not be susceptible to H3N8 virus infection (21 available strains were detected in chickens, and only 5 of the 21 were detected before 2022) (Figure 2B). However, the infection and replication of wild bird H3N8 viruses in chickens have not been investigated in previous studies. In this study, WD/W1895/17, WD/W6275/19 and GL/W1518/21 were further tested to evaluate their replication and transmission abilities in chickens. The serological tests prior to infection indicated that the farmed chickens had high H9 HI antibody titers but low prevailing H5 (Re-13, Re-14) and H7 (Re-4) HI antibody titers (Table S3). Viral titers of samples of each organ from the inoculated chickens were tested at 3 days post inoculation (dpi). Interestingly, only very low viral titers were detected in the trachea, pancreas and intestine, and no virus was detected in the lung, liver, spleen, kidney, rectum or bursa of Fabricius (Figure 8A). Viral titers of oropharyngeal and cloacal swab samples from the inoculated and contact chickens were also detected at the indicated time points. Unlike those in ducks, the three representative viruses displayed very limited transmissibility in five pairs of chickens. In the WD/W1895/17 and GL/W1518/21 groups, virus was detected in four inoculated chickens and in three contact chickens (Figure 8B and D). In the WD/W6275/19 group, virus was detected in three inoculated chickens and in one contact chicken on day 11 postinoculation (pi) (Figure 8C). Notably, the viral titers of oropharyngeal and cloacal swab samples of the positive inoculated and contact chickens were low, and the viral shedding period of the chickens was short compared to that of ducks (Figure 8B–D). Importantly, H3N8 virus infection hardly induced HI antibody production in the serum in all five inoculated or contact chickens (Table S4). The chicken experiments indicated that farmed chickens are not susceptible to infection with representative wild bird-derived H3N8 viruses.

### Commercial pigeons were not susceptible to representative wild bird H3N8 viruses

Free-ranging domestic pigeons can come in contact with both wild birds and domestic ducks and chickens. As a result, we tested the replication and transmission of the representative H3N8 viruses in commercial pigeons. The serological tests prior to infection indicated that the HI antibody of pigeons could not bind to H5 (Re-13, Re-14) and H7 (Re-4) viruses, and these birds had very low H9 HI antibody titers in their serum (Table S3). Virus was not detected in all the collected organs except for one lung sample in the WD/W1895/17 group, which was positive but had a very low viral titer (Figure 8E). WD/W6275/19 virus was detected in the liver and kidney in one pigeon, while GL/W1518/21 was detected in the pancreas and rectum in two pigeons (Figure 8E). Although virus was detected in the oropharyngeal or cloacal swabs of five inoculated pigeons and four contact pigeons in the WD/W1895/17 group, viral shedding was not persistent, and the viral titers were relatively low (Figure 8F). Similarly, the other two viruses (WD/W6275/19 and GL/W1518/21) showed limited viral shedding and low transmissibility in pigeons (Figure 8G and H). Interestingly, we did not detect any HI antibody in the serum of the inoculated and contact pigeons at 10, 15, and 21 dpi, suggesting that inoculation with the representative H3N8 viruses did not stimulate the production of specific antibodies in pigeons (Table S4). These infection studies showed that wild bird-origin H3N8 viruses are not able to replicate in pigeons.

### Thermal stability and neuraminidase activities of the H3N8 viruses

Thermal stability is important for the survival of influenza viruses in nature and is also reported to be correlated with the transmissibility of some H5N1 viruses [40]. We therefore compared the thermal stability of the three representative viruses. We found that the three viruses reduced viral titers by one unit and 3–4 logs after 4 h of treatment at 50°C, which suggested that these viruses were thermally stable (Figure S5). NA proteins promote progeny virion release by cleaving sialic acids on the host cell surface, contributing to virus replication in host cells [41,42]. Accordingly, we further tested the enzymatic activities of NA of the three selected H3N8 viruses. We found that the NA activities of WD/W6275/19 and GL/W1518/21 were higher than those of WD/W1895/17 (Figure S6).

## Discussion

In recent decades, emerging novel animal influenza viruses and other zoonoses have posed major challenges to the global commercial poultry industry and public health [5,43,44]. However, H3N8 avian influenza viruses have been ignored due to their low prevalence in chickens and the low risk to public health, although it has become one of the predominant strains in migratory waterfowl and domestic ducks. Recent human infections with H3N8 viruses in China have promoted interest in and concern about the evolution and cross-species transmission risk of these viruses. In this study, we performed a detailed analysis of the global distribution of H3N8 viruses in different hosts according to deposited sequence data and our surveillance results in eastern China between 2017 and 2021. The summarized results indicated that migratory ducks are the primary natural reservoirs of H3N8 viruses. Here, a total of 162 viruses belonging to 28 subtypes were identified and isolated from 15,899 wild bird samples, which suggested that wild birds are natural reservoirs of different subtypes of avian influenza viruses and play a key role in the maintenance and evolution of such viruses.

H3N8 viruses have been detected in at least 64 kinds of wild birds and have evolved into several phylogenetic lineages, whereas only a few strains were detected in chickens in recent years. The H3N8 viruses analyzed in this study shared similar sequence identities and clustered into the same lineages with some strains isolated from Europe, North America and Africa, suggesting that H3N8 viruses can be transmitted globally with the migration of their natural reservoirs. We also found that these H3N8 viruses have undergone complicated reassortment with circulating H5N3, H7N4, H9N2, H10N4 and H10N8-like viruses isolated from Yellow River Delta wetlands [21,33,34]. Of note, all the H3N8 viruses detected from chicken and humans in China in 2022 share HA and NA surface genes similar to those of duck- and wild bird-origin strains but bear an internal gene constellation from chicken H9N2 viruses [29,31]. The predominantly prevalent H9N2 viruses have been proven to be ideal internal gene donors for the emerged reassortants, including H7N9, H10N3, and H10N8 viruses [8,13,45,46]. The complicated epidemiology and ecology of avian influenza viruses at the interface of waterfowl and terrestrial birds could have facilitated the emergence of novel H3N8 reassortants in chickens. Therefore, monitoring AIVs in wild and domestic waterfowl and controlling H9N2 viruses in domestic birds will contribute to early warning and reduction in the occurrence of natural avian influenza reassortants.

Key amino acid substitutions and the conversion of receptor binding specificity have contributed to the virulence and transmissibility of AIVs in mammals. In the first human infection case, a 4-year-old boy was reported to be infected with H3N8 virus and developed severe acute respiratory distress syndrome in a short period of time, implying that the internal gene constellation of the H9N2 virus may contribute to increased virulence of the H3N8 virus isolated from human [10]. Yang et al. compared the genetic differences of the two human isolates and the chicken isolates and found that the first human strain A/Henan/4-10/2022 acquired the PB2 E627K mutation compared with the chicken ancestor viruses [29]. Zhang et al. found that some H3N8 viruses isolated from wild birds could be transmitted efficiently among guinea pigs and that the PB1 S524G mutation conferred increased virulence in mice and airborne transmissibility in ferrets [28]. Li et al. found that wild bird-derived H3N8 exhibited good adaptation in mice and induced significant weight loss in mice [47]. In this study, mutations involved in the enhanced virulence of the viruses in mammals, including 207 K and 436Y in PB1, 515 T in PA, and 30D and 215A in M1, were observed in the 21 H3N8 isolates. Animal studies indicated that some H3N8 isolates can replicate efficiently in lung and nasal turbinate tissue and cause weight loss in mice without prior adaptation. The change in receptor binding specificity from avian-type to human-type is a primary determinant for efficient AIV transmission to mammals or humans. Several publications previously reported that naturally isolated H3N8 viruses from wild birds exhibited dual receptor-binding profiles [28,47,48]. Yang et al. tested the receptor binding properties of one human H3N8 isolate and five chicken isolates by direct binding assays with SAα2–3Gal and SAα2–6Gal sialylglycopolymers and found that all the tested viruses could bind to both avian-type and human-type receptors. Residues N193, W222 and S227 might contribute to the dual receptor-binding properties of chicken H3N8 viruses [29]. The wild bird-origin H3N8 viruses in this study had key residues in the receptor-binding region of HA similar to those of the human and chicken isolates, including N193, W222, Q226, S227 and G228. Residues 155 T and 160A, which confer the enhanced receptor-binding property of avian influenza viruses, were observed in the 21 H3N8 viruses [8,35]. These residues in the receptor-binding regions of HA may collectively contribute to the dual receptor-binding profiles of wild bird H3N8 viruses. The transmission of these H3N8 viruses in mammals, such as guinea pigs and ferrets, needs to be evaluated in further studies.

Ducks are primary reservoirs of different AIVs and play a key role in cross-species transmission at the waterfowl and terrestrial bird or mammal interface [49]. Our previous study found that H10N4 and H10N8 viruses isolated from wild birds could replicate and be efficiently transmitted to ducks, but they did not induce high HI antibody production in ducks [21]. In this study, we found that wild bird-derived H3N8 viruses replicated poorly in DEF cells in vitro but replicated efficiently in ducks, especially in the enteric canal of ducks. Moreover, these H3N8 viruses exhibited highly efficient transmissibility between ducks and persistent viral shedding. However, these viruses did not induce the production of high titers of HI antibody in ducks. Chickens are susceptible to many strains of AIVs, including highly pathogenic H5 and H7 viruses and low-pathogenic H4, H6, H7, H9, and H10 viruses [19,34,45,46]. However, some wild bird strains, such as H16, have shown poor adaptation in chickens [50,51]. Here, we found that unlike ducks, commercial chickens were not susceptible to these H3N8 viruses. Importantly, seroconversion in chickens was detected in only a few inoculated birds and none of the contact chickens. Pigeons are not susceptible to AIVs, but several studies have found that some strains can replicate in pigeons [52,53]. Here, we found that pigeons were not susceptible to the representative wild bird H3N8 viruses and that the viruses did not induce the production of HI antibodies in inoculated pigeons. The experimental studies revealed that ducks but not chickens or pigeons were susceptible to the representative wild bird-derived H3N8 viruses. Different biological characteristics among ducks, chickens and pigeons can explain why the H3N8 virus sequences deposited in GenBank and GISAID were mainly detected in wild birds (mainly migratory ducks) and domestic ducks, while only a few strain sequences were detected in chickens and pigeons.

In summary, we described the distribution of H3N8 viruses and characterized the genetic and biological properties of H3N8 viruses isolated from wild birds in a wetland in eastern China. Our findings emphasize that active surveillance in migratory birds and domestic ducks will contribute to early detection of the emergence and evolution of AIVs in waterfowl and potential threats to the commercial poultry industry and human health.

## Materials and methods

### Ethics statement and facility

The animal studies were carried out in strict accordance with the recommendations in the Guide for the Care and Use of Laboratory Animals of the Ministry of Science and Technology of the People’s Republic of China. The protocols for chicken, duck, pigeon and mouse studies were approved by the Committee on the Ethics of Animal Experiments of Liaocheng University. The ethics approval numbers are CK-INFEC-2022-03 (chicken); DK-INFEC-2022-01 (duck); PG-INFEC-2022-03 (pigeon); MC-INFEC-2022-05 (mice). All experiments with H3N8 viruses were conducted in an animal biosafety level 2 (ABSL-2) facility. The animals used in this study were placed in a biological safety isolator.

### Experimental animals

Six-week-old specific pathogen-free (SPF) female BALB/c mice were purchased from Jinan Pengyue Experimental Animal Breeding Co., Ltd. (Shandong, China). Three-week-old SPF ducks were purchased from Shandong Healthtech Laboratory Animal Breeding Co., Ltd. Forty-five-day-old commercial layer chickens and 30-day-old commercial pigeons were purchased from local poultry farms.

### Data acquisition from public databases

The HA sequences of H3N1, H3N2, H3N3, H3N4, H3N5, H3N6, H3N7, H3N8, and H3N9 subtype viruses that were detected in avian and mammalian species (human strains are not summarized in this study) were downloaded from the Influenza Virus Database of NCBI (https://www.ncbi.nlm.nih.gov/genomes/FLU/Database/nph-select.cgi?go=database) and GISAID (https://www.gisaid.org), respectively. The HA sequences of the same subtype downloaded from NCBI and GISAID were input into MEGA 11, and redundant sequences with the same ID were removed. Then, the number of HA sequences of each H3 subtype was summarized and visualized by Prism 9. The HA sequences of animal H3N8 strains collected each year were downloaded from NCBI and GISAID and input to MEGA 11 to delete the overlapping sequences. The number of HA sequences of the H3N8 viruses in different hosts was then summarized. The H3N8 viruses detected from avian species were classified into specific bird groups according to the virus isolation information. The available H3 viruses in public databases whose NA subtypes were not identified were not summarized in this study. The public viral sequences available in the NCBI and GISAID databases were updated to November 25, 2022.

### Sample collection, virus identification and isolation

The sampling sites were located in the Yellow River Delta wetland, which is an important habitat for migratory birds along the East Asian-Australasian (EAA) migratory flyway in eastern China. The species of the wild birds were first determined by binoculars in the habitats. Fresh fecal droppings of wild ducks, gulls and other wild birds were collected and then placed into 2 ml of minimal essential medium supplemented with penicillin and streptomycin. Positive fecal samples were identified by PCR with specific M and HA (H5, H7) primers [54,55]. The suspected H5- or H7-positive samples were transferred to an enhanced ABSL-3 facility in the Harbin Veterinary Research Institute of Chinese Academy of Agricultural Sciences for further virus identification and isolation, while the remaining suspected positive samples were injected into 10-day-old embryonated chicken eggs to isolate the viruses in an ABSL-2 laboratory at Liaocheng University. The isolated viruses were stored in a −80°C freezer.

### Genetic and molecular analysis

Genome sequencing of the H3N8 viruses was performed on an Applied Biosystems DNA Analyzer (3500xL Genetic Analyzer, USA) at Harbin Veterinary Research Institute of Chinese Academy of Agricultural Sciences (HVRI, CAAS). The sequence data were compiled with the SEQMAN programme (DNASTAR, Madison, WI) according to the reference sequences. The molecular markers in each segment were identified with the MegAlign programme (DNASTAR, Madison, WI).

### Phylogenetic analysis

The sequences of the 21 viruses in this study and the downloaded full-length sequences from databases were imported into Mega 11.0 for aggregation to obtain a single file. Multiple sequence alignment was performed by mafft software (v7.505) [56]. The repeated sequences and partial sequences were deleted, then the reference viruses were picked according to their host, collcetion region and collection date as shown in the phylogenetic tree. The best-fit nucleotide substitution model was selected using IQ-tree (v1.6.12) [57]. The branch support value in maximum likelihood (ML)-based trees was assessed by the ultrafast bootstrap approximation test and Shimodaira-Hasegawa approximate likelihood ratio (SH-aLRT) test. The ML trees of the six internal genes (PB2, PB1, PA, NP, M, NS) were visualized and embellished by FigTree (v1.4.4). The SH-aLRT support (%)/ultrafast bootstrap support (%) are shown at the nodes.

Markov chain Monte Carlo (MCMC) trees with molecular clocks were constructed using BEAST (v1.10) software to study the evolutionary history of H3N8 viruses in wild birds [58]. All the picked sequences were filtered from TempEst by assessing whether there was sufficient temporal signal in our data to proceed with phylogenetic molecular clock analysis. The path-sampling and stepping-stone estimation approaches were used to assess the best fitting clock model through marginal likelihood estimation. The best-fit nucleotide substitution model was selected using IQ-tree. The GTR + F + I + G4 distributed rate variation among sites in the nucleotide substitution model was selected, and MCMC chains were run for 2 × 10^9^ iterations and sampled every 10,000 steps to generate a BEAST file (Figure 3A and B). The TN + F + G4 nucleotide substitution model was selected, and MCMC chains were run for 3 × 10^8^ iterations and sampled every 1000 steps to generate a BEAST file. The HKY + F + G4 substitution model was selected, and MCMC chains were run for 5 × 10^8^ iterations and sampled every 10,000 steps to generate a BEAST file (Figure 4). Both HA and NA genes were chosen with an uncorrelated lognormal relaxed molecular clock and a Bayesian skyline coalescent tree prior. Tracer (v1.7.1) was used to observe whether the parameters converged (effective sample size values ≥200). The MCMC tree files were obtained using TreeAnnotator software, with 10% burn-in FigTree (v1.4.4) used to generate the MCMC trees with a time scale.

### Receptor binding assay

The solid-phase direct binding assay to test the receptor binding properties of the viruses has been described previously [21,34]. A specific chicken anti-H3 polyclonal antibody was used to detect H3N8 viruses. Dose–response curves of virus binding to glycopolymers were analyzed by using a single-site binding algorithm and curve fitting by GraphPad Prism 8 to determine the associated constant (Ka) values. Each value is presented as the mean ± standard deviation (SD) of three independent experiments, each of which was performed in triplicate.

### Antigenic analysis

The chicken, duck and pigeon antisera used in this study were generated with birds that were inoculated with 10^6^ EID_50_ of the tested viruses in a volume of 200 µl. Antigenic analysis was performed by using the HI assay with 1% chicken erythrocytes.

### Multicycle growth kinetics

Chicken embryo fibroblast (CEF) cells were obtained from 10-day-old SPF chicken embryonated eggs. Duck embryo fibroblast (DEF) cells were obtained from 12-day-old SPF duck embryonated eggs. Madin-Darby canine kidney (MDCK) and human lung adenocarcinoma epithelial (A549) cells were purchased from the Cell Resource Center of the Shanghai Institute of Life Sciences and preserved by our laboratory. MDCK, CEF and DEF cells were grown in Dulbecco’s modified Eagle’s medium (DMEM) containing 10% fetal bovine serum (FBS) and antibiotics. A549 cell lines were grown in an F-12 K nutrient mixture containing 10% FBS and antibiotics. All cells were cultured at 37°C with 5% CO_2_. Monolayer cells grown in 24-well plates were inoculated with 10^5^ EID_50_ of the virus in a volume of 200 µl. One hour later, the supernatant was discarded, the wells were washed with phosphate-buffered saline (PBS) three times, and 500 µl of OPTI-MEM (GIBCO) was then added to the wells. OPTI-MEM containing 0.5 µg/ml trypsine-TPCK was used to assist in the infection of MDCK and A549 cells with the low-pathogenicity H3N8 virus. The supernatant was collected at 12, 24, 48 and 72 h postinoculation (hpi) and then titrated in eggs. The growth data shown are the average results of three independent experiments.

### Animal experiments

#### Mice

Six-week-old female SPF mice (eight animals in each group) were inoculated with 10^6^ EID_50_ of the virus in a volume of 50 µl. Three mice were euthanized on day 3 pi, and nasal turbinate, lung, spleen, kidney, and brain tissues were collected for viral titration in eggs. The lungs of three mice were fixed in 10% formalin and then stained with hematoxylin and eosin (HE) for histological analysis. The remaining five mice were monitored daily for 14 days for weight loss and survival. Mice inoculated with PBS were established as a control group and used to observe body weight changes.

#### Chickens

Forty-five-day-old commercial layer chickens were used in the infection study. Oropharyngeal and cloacal swabs of the commercial chickens were collected to detect AIVs and Newcastle disease virus (NDV) by both PCR and viral titration in eggs [54]. The HI antibody against the H3N8 virus in chicken serum was also detected by the HI assay before the infection study. The chickens that tested negative were divided into three groups to analyze the replication of WD/1895/17, WD/11221/19, and GL/W1518/21 viruses in chickens. Three chickens from each group were inoculated with 10^6^ EID_50_ of the virus in a volume of 200 µl. Brain, tracheal, lung, liver, spleen, pancreatic, kidney, intestinal, rectal, and bursa of Fabricius tissue samples of the chickens were collected for viral titration in eggs at day 3 pi.

For the transmission study, five chickens from each group were inoculated with 10^6^ EID_50_ of the virus in a volume of 200 µl. Another five naive chickens were placed into the same isolator at 24 hpi. Oropharyngeal and cloacal swabs of the chickens were collected on days 1, 3, 5, 7, 9, and 11 pi. The viral titers of the swabs were determined in eggs. Chicken serum was collected on days 10, 15, and 21 pi, and the antibody titer was determined by the HI test. The chickens were then euthanized on day 21 pi.

#### Ducks

Twenty-seven three-week-old SPF ducks were divided into three groups to analyze the replication of WD/1895/17, WD/11221/19, and GL/W1518/21 viruses in ducks. Three ducks from each group were inoculated with 10^6^ EID_50_ of the virus in a volume of 200 µl. Brain, tracheal, lung, liver, spleen, pancreatic, kidney, intestinal, rectal, and bursa of Fabricius tissue samples of the ducks were collected for viral titration in chicken eggs at day 3 pi.

For the transmission study, three ducks from each group were inoculated with 10^6^ EID_50_ of the virus in a volume of 200 µl. Another three naive ducks were placed into the same isolator at 24 hpi. Oropharyngeal and cloacal swabs of the ducks were collected on days 1, 3, 5, 7, 9, and 11 pi. The viral titers of the swabs were titrated in eggs. Duck serum was collected on days 10, 15, and 21 pi, and the antibody titer was determined by the HI test. The ducks were then euthanized on day 21 pi.

#### Pigeons

Four-week-old commercial pigeons were purchased from a local poultry farm. Oropharyngeal and cloacal swabs were collected to detect AIVs and NDV by qPCR and for virus isolation. Pigeon serum was collected to detect the HI antibody against H3N8 viruses by the HI assay before the infection study. The methods for virus inoculation, collection and virus titration in the organs, collection of oropharyngeal and cloacal swabs, collection of pigeon serum, and the HI test were the same as those used for the chicken study, as described above.

### Heat stability test

Test viruses (32 HA units in PBS) were incubated at 50°C for 30, 60, 120, 180, and 240 min. Hemagglutination activity was then determined by hemagglutination assays using 0.5% chicken red blood cells, and the virus infectivity (EID_50_) was determined in 10-day-old chicken embryos. All experiments were repeated in triplicate.

### Neuraminidase activity assay

The neuraminidase activity assay has been described previously [34]. In brief, the test viruses were serially diluted 2-fold from 1 × 10^7^ EID_50_/ml to 9.8 × 10^3^ EID_50_/ml. Then, 50 μl of 200 μM substrate 2′-(4-methylumbelliferyl)-α-d-N-acetylneuraminic acid (MUNANA) was mixed with 50 μl of the virus dilution and incubated at 37°C for 60 min, and the reaction was stopped by adding 100 μl of 0.2 M Na_2_CO_3_. Finally, fluorescence was measured at excitation and emission wavelengths of 365 nm and 450 nm, respectively. The neuraminidase activity assay was performed in triplicate.

## Supplementary Material

Supplemental Material
