# Supplementary material for: Prevalence, evolution, replication and transmission of H3N8 avian influenza viruses isolated from migratory birds in eastern China from 2017 to 2021
Source: Emerg Microbes Infect. 2023 Mar 13;12(1):2184178. doi: 10.1080/22221751.2023.2184178 (PMC10013397; doi:10.1080/22221751.2023.2184178)
Supplement: Supplemental Material [file TEMI_A_2184178_SM5603.pdf]

Figure S1

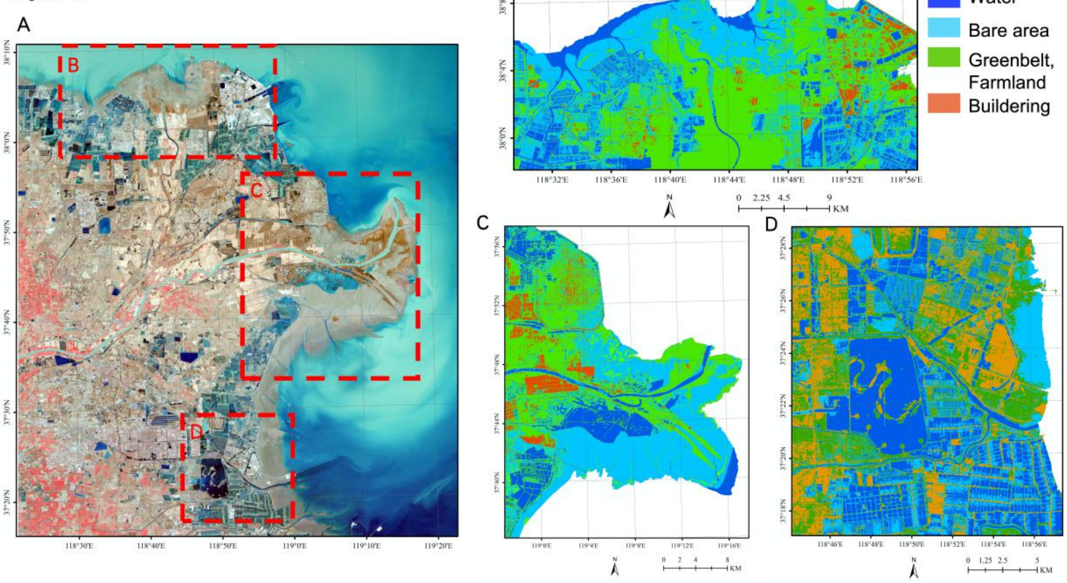

Figure S1. Sampling area at Yellow River Delta wetland from 2017 to 2021.

Figure S2 A
PB2

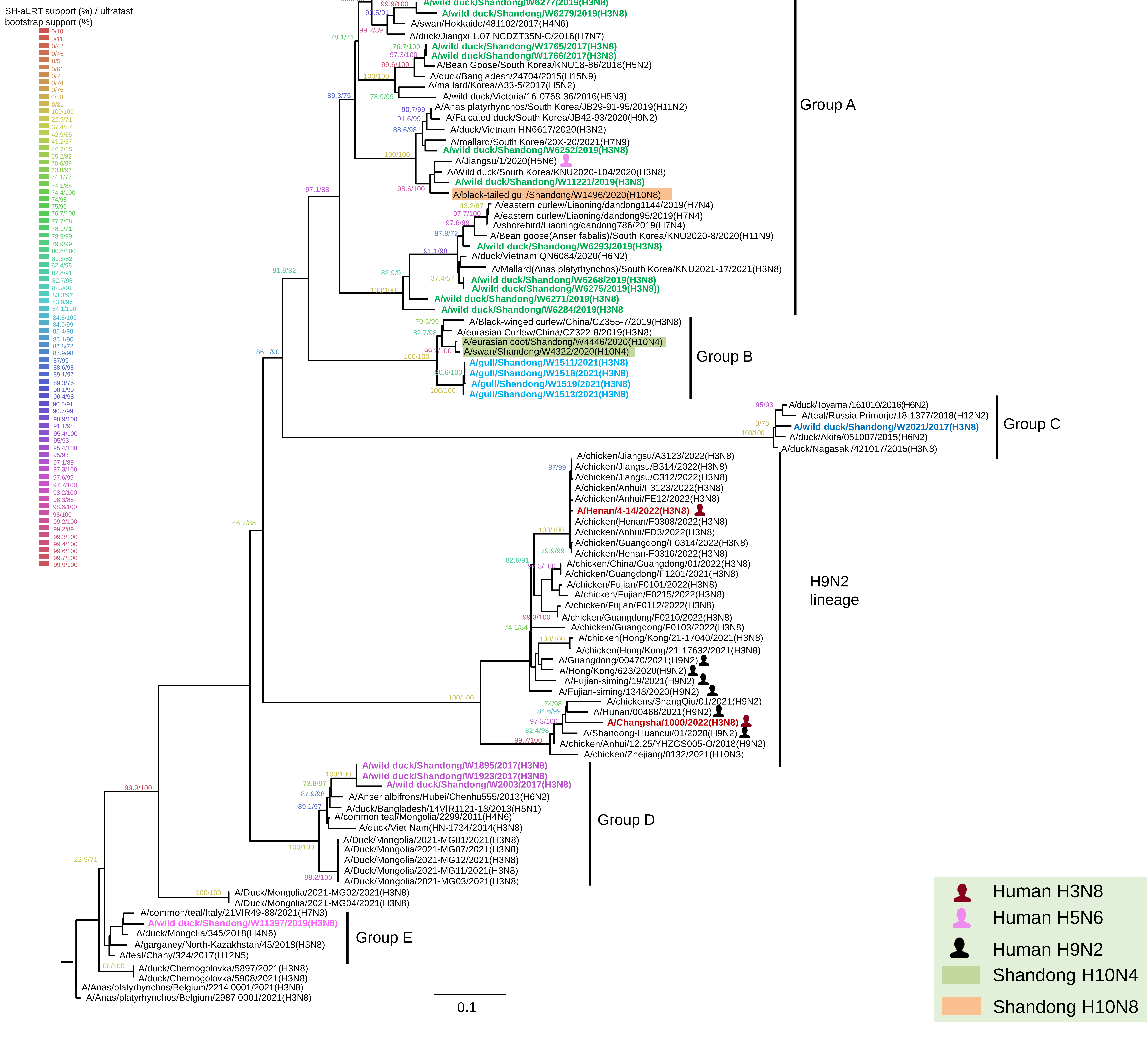

Figure S2 B
PB1

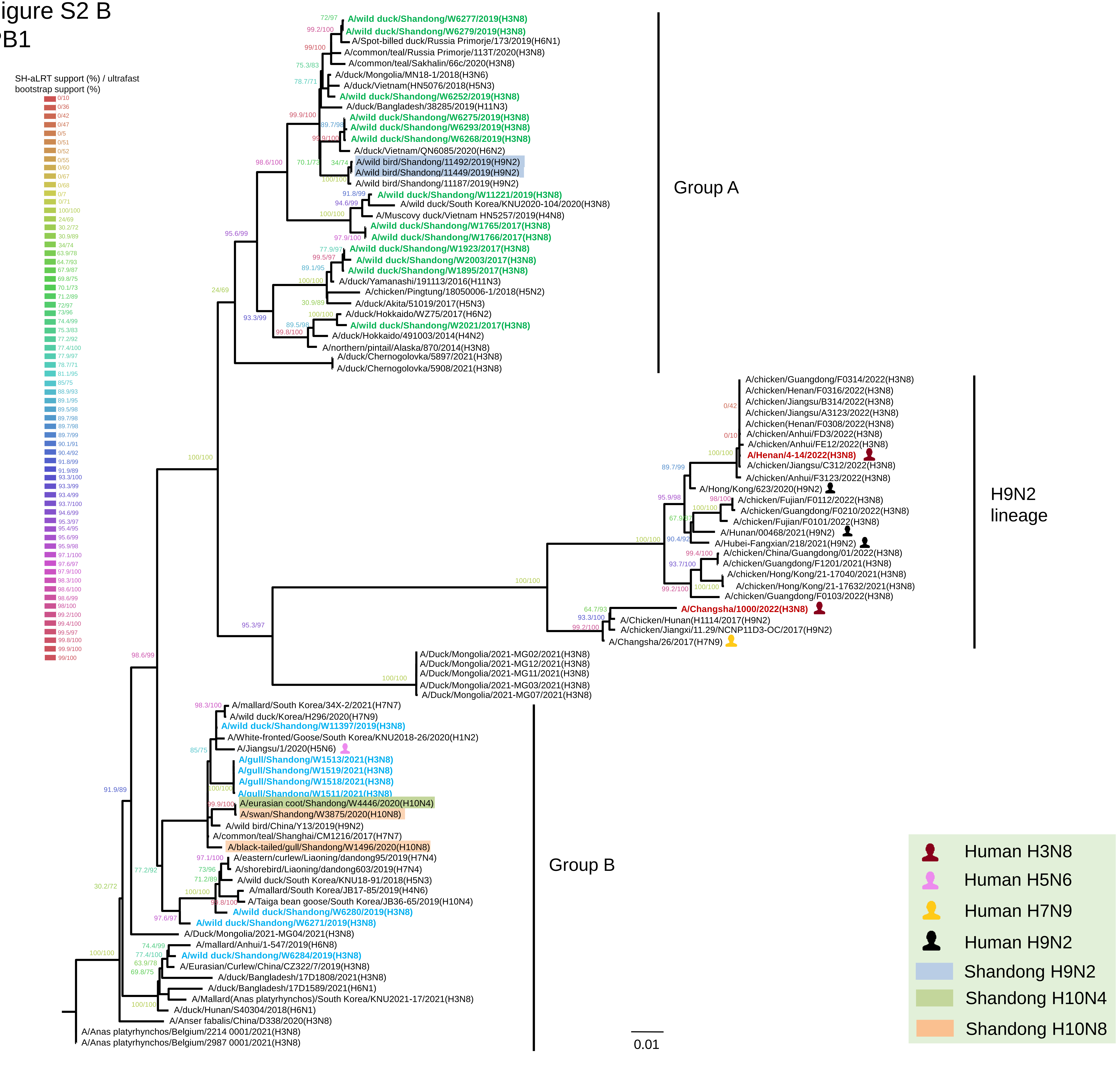

Figure S2 C
PA

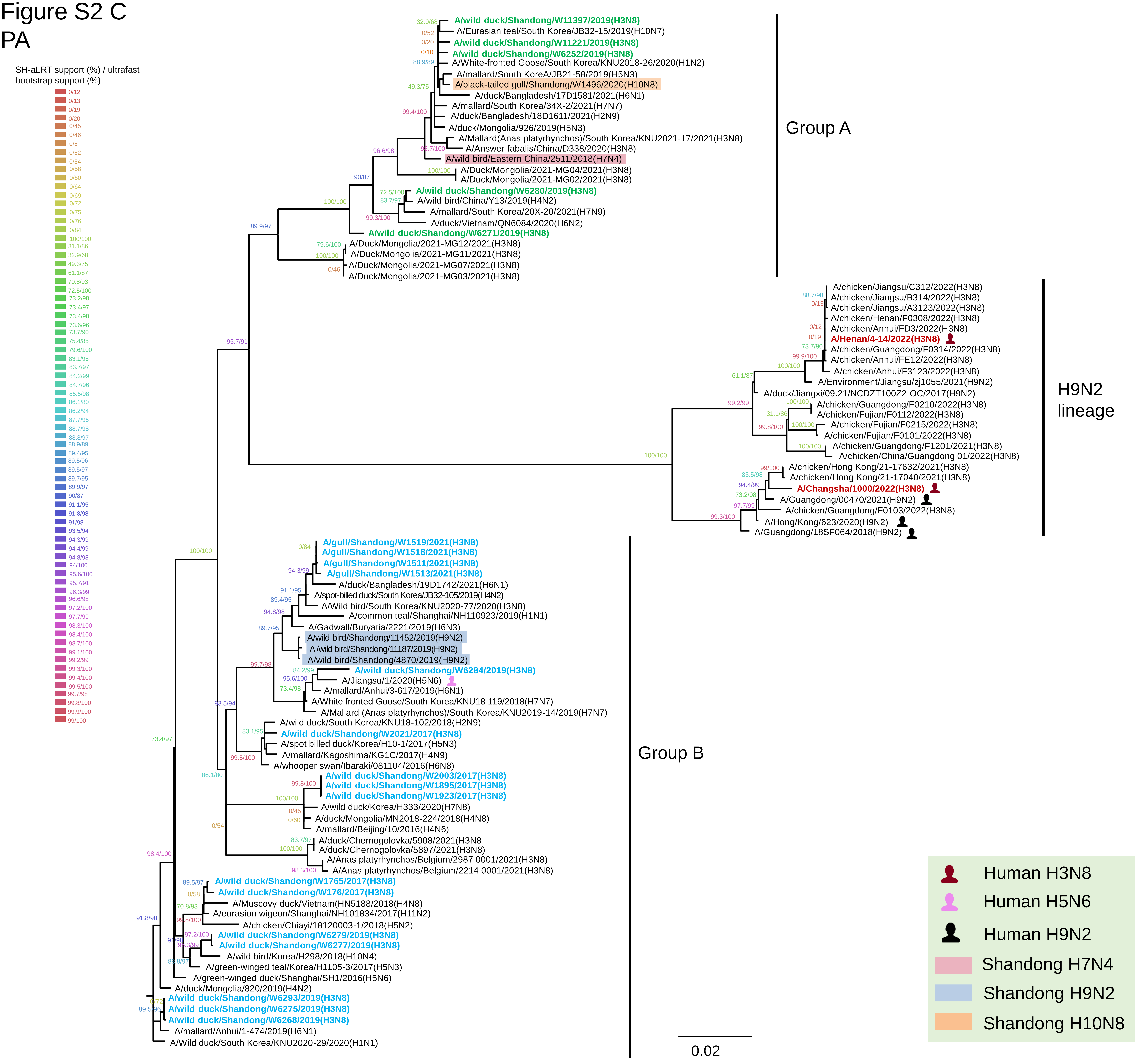

Figure S2 D
NP

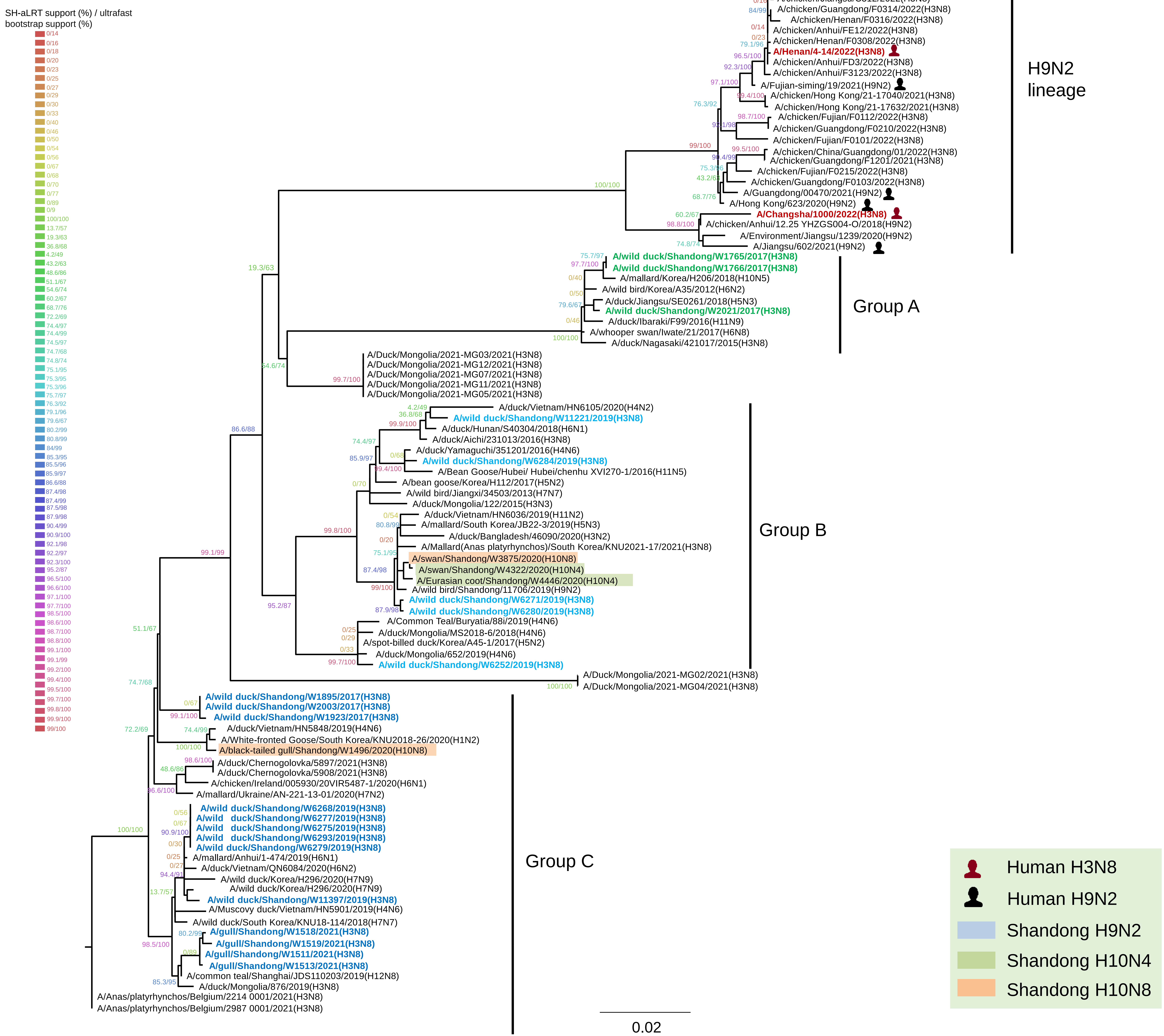

Figure S2 E
M

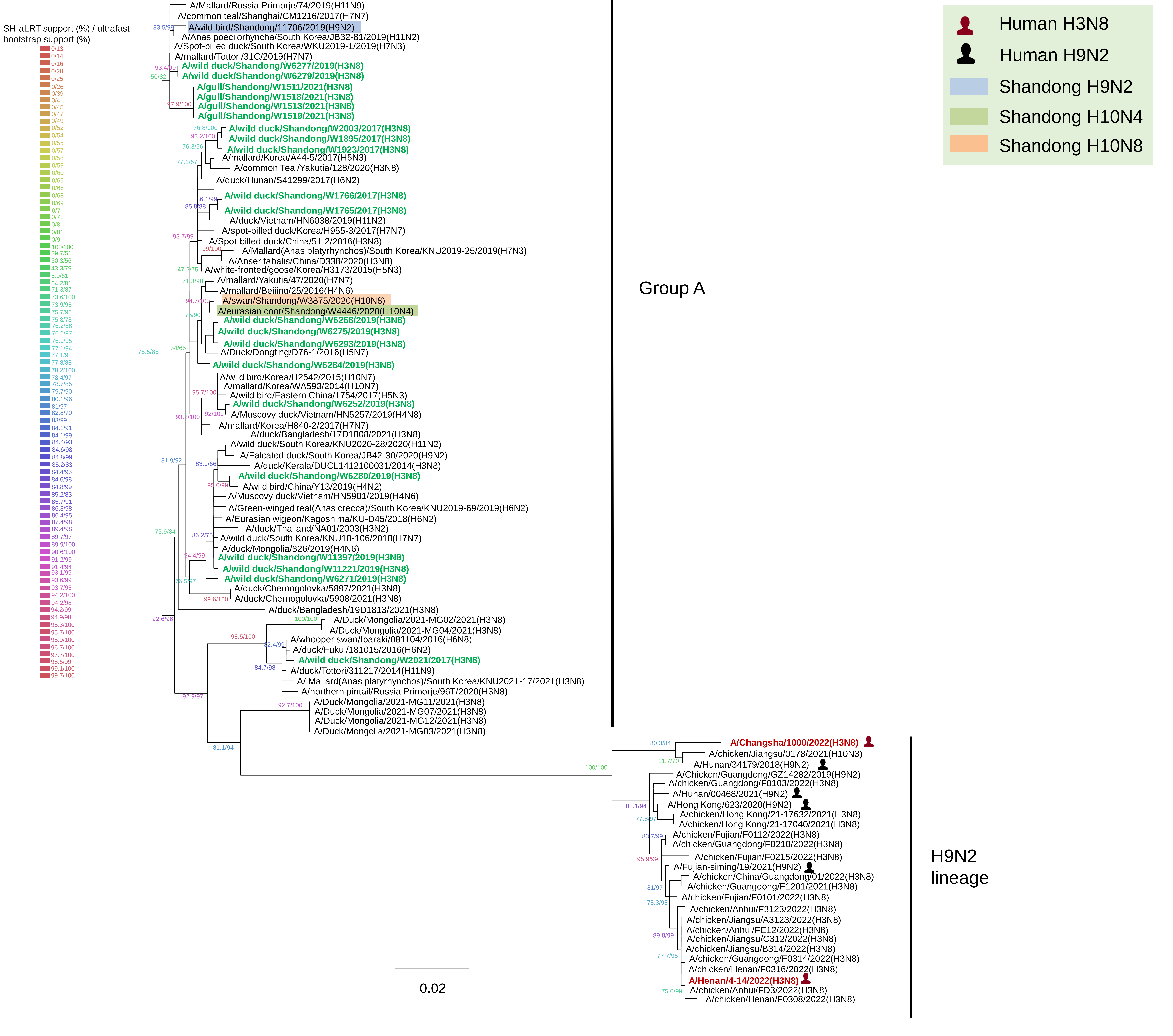
SH-aLRT support (%) / ultrafast bootstrap support (%)
Human H3N8
Human H9N2
Shandong H9N2
Shandong H10N4
Shandong H10N8
A/Anas/platyrhynchos/Belgium/2214 0001/2021(H3N8)
A/Anas/platyrhynchos/Belgium/2987 0001/2021(H3N8)
A/Mallard/Russia Primorje/74/2019(H11N9)
A/common teal/Shanghai/CM1216/2017(H7N7)
A/wild bird/Shandong/11706/2019(H9N2)
A/Anas poecilorhyncha/South Korea/JB32-81/2019(H11N2)
A/Spot-billed duck/South Korea/WKU2019-1/2019(H7N3)
A/mallard/Tottori/31C/2019(H7N7)
A/wild duck/Shandong/W6277/2019(H3N8)
A/wild duck/Shandong/W6279/2019(H3N8)
A/gull/Shandong/W1511/2021(H3N8)
A/gull/Shandong/W1518/2021(H3N8)
A/gull/Shandong/W1513/2021(H3N8)
A/gull/Shandong/W1519/2021(H3N8)
A/wild duck/Shandong/W2003/2017(H3N8)
A/wild duck/Shandong/W1895/2017(H3N8)
A/wild duck/Shandong/W1923/2017(H3N8)
A/mallard/Korea/A44-5/2017(H5N3)
A/common Teal/Yakutia/128/2020(H3N8)
A/duck/Hunan/S41299/2017(H6N2)
A/wild duck/Shandong/W1766/2017(H3N8)
A/wild duck/Shandong/W1765/2017(H3N8)
A/duck/Vietnam/HN6038/2019(H11N2)
A/spot-billed duck/Korea/H955-3/2017(H7N7)
A/Spot-billed duck/China/51-2/2016(H3N8)
A/Mallard(Anas platyrhynchos)/South Korea/KNU2019-25/2019(H7N3)
A/Anser fabalis/China/D338/2020(H3N8)
A/white-fronted/goose/Korea/H3173/2015(H5N3)
A/mallard/Yakutia/47/2020(H7N7)
A/mallard/Beijing/25/2016(H4N6)
A/swan/Shandong/W3875/2020(H10N8)
A/eurasian coot/Shandong/W4446/2020(H10N4)
A/wild duck/Shandong/W6268/2019(H3N8)
A/wild duck/Shandong/W6275/2019(H3N8)
A/wild duck/Shandong/W6293/2019(H3N8)
A/Duck/Dongting/D76-1/2016(H5N7)
A/wild duck/Shandong/W6284/2019(H3N8)
A/wild bird/Korea/H2542/2015(H10N7)
A/mallard/Korea/WA593/2014(H10N7)
A/wild bird/Eastern China/1754/2017(H5N3)
A/wild duck/Shandong/W6252/2019(H3N8)
A/Muscovy duck/Vietnam/HN5257/2019(H4N8)
A/mallard/Korea/H840-2/2017(H7N7)
A/duck/Bangladesh/17D1808/2021(H3N8)
A/wild duck/South Korea/KNU2020-28/2020(H11N2)
A/Falcated duck/South Korea/JB42-30/2020(H9N2)
A/duck/Kerala/DUCL1412100031/2014(H3N8)
A/wild duck/Shandong/W6280/2019(H3N8)
A/wild bird/China/Y13/2019(H4N2)
A/Muscovy duck/Vietnam/HN5901/2019(H4N6)
A/Green-winged teal(Anas crecca)/South Korea/KNU2019-69/2019(H6N2)
A/Eurasian wigeon/Kagoshima/KU-D45/2018(H6N2)
A/duck/Thailand/NA01/2003(H3N2)
A/wild duck/South Korea/KNU18-106/2018(H7N7)
A/duck/Mongolia/826/2019(H4N6)
A/wild duck/Shandong/W11397/2019(H3N8)
A/wild duck/Shandong/W11221/2019(H3N8)
A/wild duck/Shandong/W6271/2019(H3N8)
A/duck/Chernogolovka/5897/2021(H3N8)
A/duck/Chernogolovka/5908/2021(H3N8)
A/duck/Bangladesh/19D1813/2021(H3N8)
A/Duck/Mongolia/2021-MG02/2021(H3N8)
A/Duck/Mongolia/2021-MG04/2021(H3N8)
A/whooper swan/Ibaraki/081104/2016(H6N8)
A/duck/Fukui/181015/2016(H6N2)
A/wild duck/Shandong/W2021/2017(H3N8)
A/duck/Tottori/311217/2014(H11N9)
A/ Mallard(Anas platyrhynchos)/South Korea/KNU2021-17/2021(H3N8)
A/northern pintail/Russia Primorje/96T/2020(H3N8)
A/Duck/Mongolia/2021-MG11/2021(H3N8)
A/Duck/Mongolia/2021-MG07/2021(H3N8)
A/Duck/Mongolia/2021-MG12/2021(H3N8)
A/Duck/Mongolia/2021-MG03/2021(H3N8)
Group A
A/Changsha/1000/2022(H3N8)
A/chicken/Jiangsu/0178/2021(H10N3)
A/Hunan/34179/2018(H9N2)
A/Chicken/Guangdong/GZ14282/2019(H9N2)
A/chicken/Guangdong/F0103/2022(H3N8)
A/Hunan/00468/2021(H9N2)
A/Hong Kong/623/2020(H9N2)
A/chicken/Hong Kong/21-17632/2021(H3N8)
A/chicken/Hong Kong/21-17040/2021(H3N8)
A/chicken/Fujian/F0112/2022(H3N8)
A/chicken/Guangdong/F0210/2022(H3N8)
A/chicken/Fujian/F0215/2022(H3N8)
A/Fujian-siming/19/2021(H9N2)
A/chicken/China/Guangdong/01/2022(H3N8)
A/chicken/Guangdong/F1201/2021(H3N8)
A/chicken/Fujian/F0101/2022(H3N8)
A/chicken/Anhui/F3123/2022(H3N8)
A/chicken/Jiangsu/A3123/2022(H3N8)
A/chicken/Anhui/FE12/2022(H3N8)
A/chicken/Jiangsu/C312/2022(H3N8)
A/chicken/Jiangsu/B314/2022(H3N8)
A/chicken/Guangdong/F0314/2022(H3N8)
A/chicken/Henan/F0316/2022(H3N8)
A/Henan/4-14/2022(H3N8)
A/chicken/Anhui/FD3/2022(H3N8)
A/chicken/Henan/F0308/2022(H3N8)
H9N2 lineage
0.02

Figure S2 F
NS

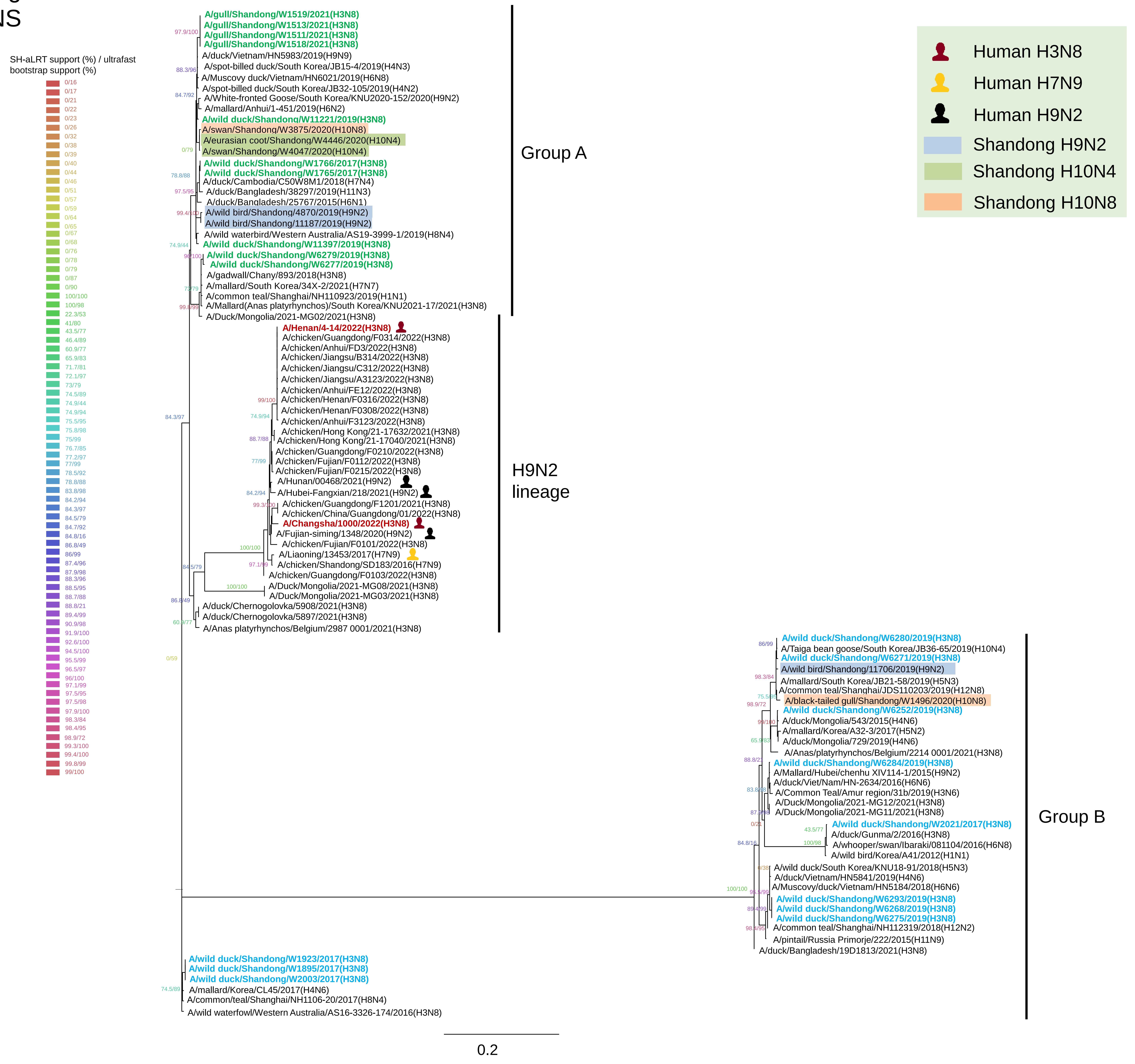

Figure S2. Phylogenetic trees of PB2 (A), PB1 (B), PA (C), NP (D), M (E), and NS (F) genes of H3N8 viruses. The sequence name colored in green, wathet blue, dark blue, purple and pink were the H3N8 viruses isolated in this study, and the sequences name in red were the human H3N8 isolates. The sequence name with black were downloaded from the databases (Genbank and GISAID).

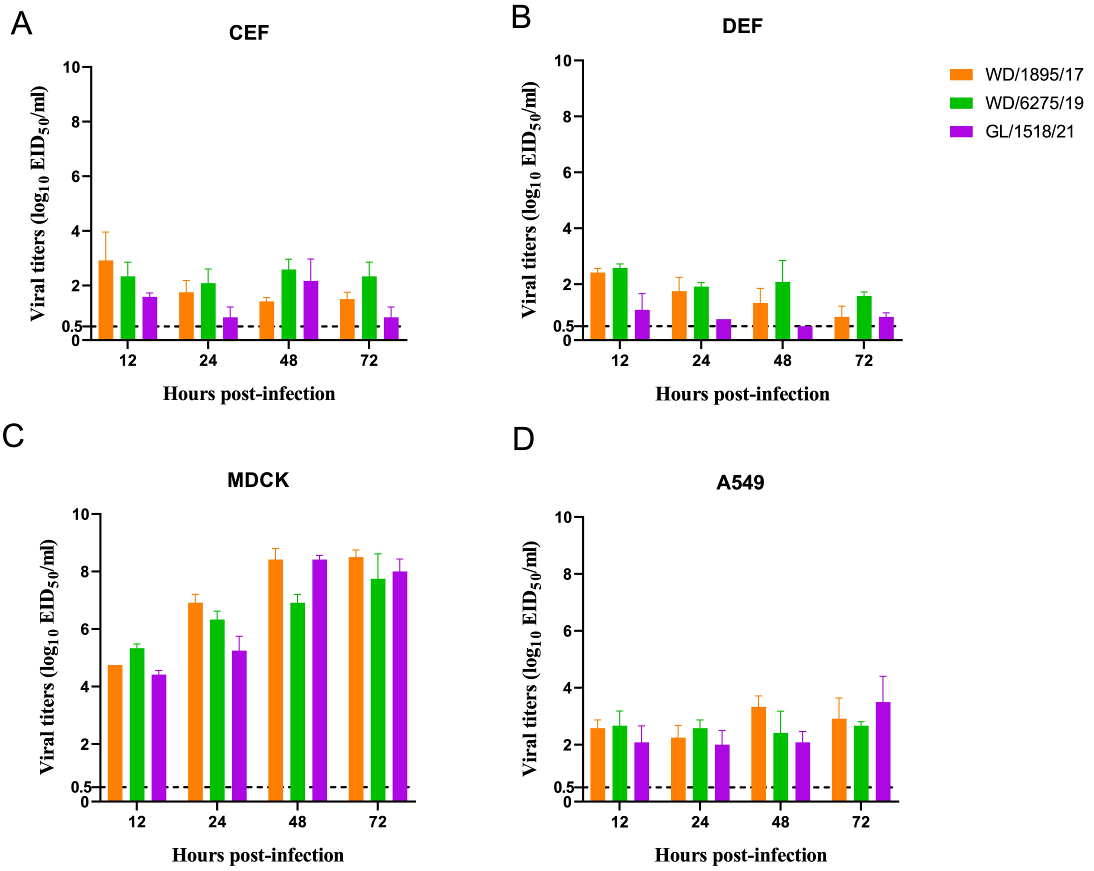

Figure S3. Growth kinetics of H3N8 viruses in chicken, duck and mammal cells. Virus growth curves in CEF, DEF, MDCK, and A549 cells. The dashed lines indicate the lower limit of detection.

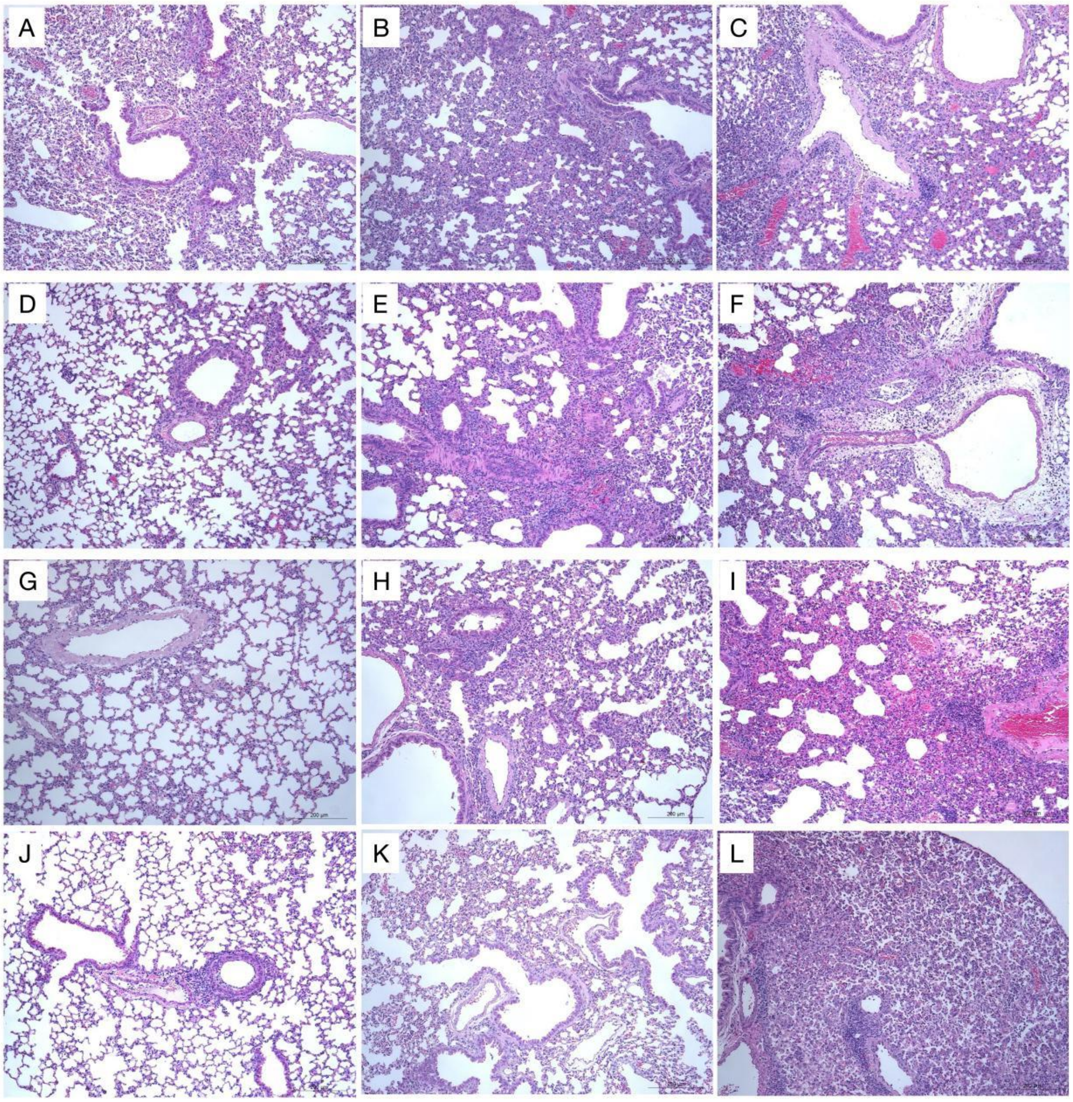

Figure S4. Pathological lesions caused by H3N8 viruses in lungs of the inoculated mice . (A) WD/W1765/17; (B) WD/W1895/17; (C) WD/W2021/17; (D) WD/W6252/19; (E) WD/W6275/19; (F) WD/W6271/19; (G) WD/W6279/19; (H) WD/W6280/19; (I) WD/W6284/19; (J) WD/W11221/19; (K) WD/W11397/19; (L) GL/W1518/21. Mice were euthanized on day 4 p.i. with 10^6 EID_{50} of test virus, and the lungs were collected for pathological studys (H&E staining). The Images (A-L) was taken at ×200 magnification.

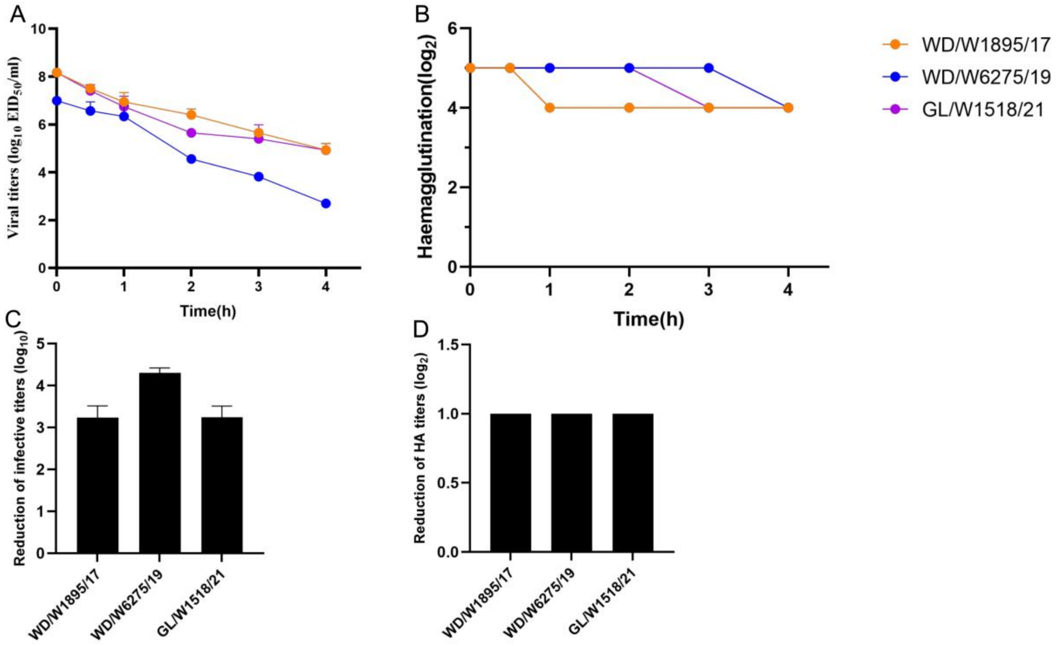

Figure S5. Effects of heat-treatment on the hemagglutination stability and infectivity of H3N8 viruses. The viruses containing 32 HAU were incubated at 50°C for the indicated times. (A) Infectivity of heat-treated viruses was determined in chicken eggs. Each point represents the mean ± standard deviation of triplicate experiments. (B) HA titers of the heat-treated viruses were determined by using hemagglutination assay with 0.5% chicken red blood cells. (C) Reduction in viral infectivity of each virus after 4 hours of heat treatment. (D) Reduction in HA titers of each virus after 4 hours of heat treatment.

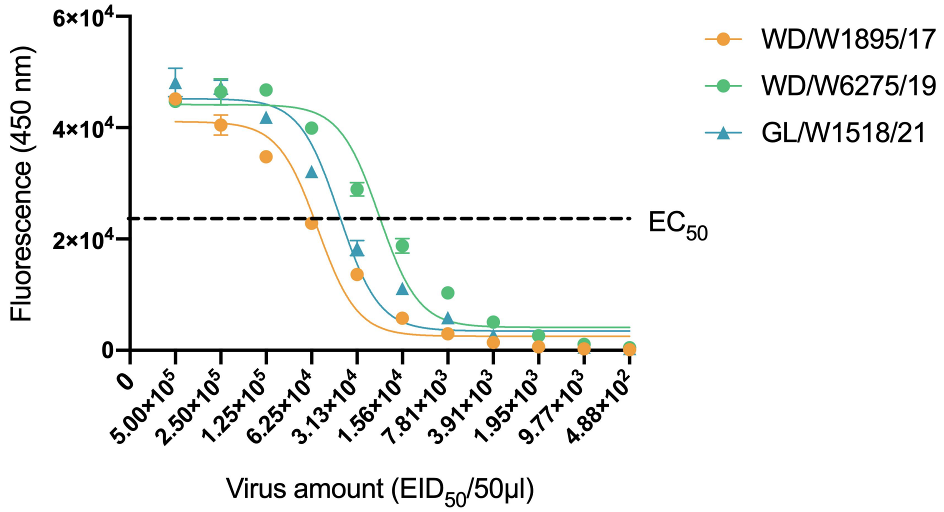

Figure S6. Neuraminidase activities of three H3N8 viruses. The neuraminidase activity was analyzed in the presence of the substrate MUNANA. The reaction was analyzed at excitation and emission wavelengths of 365 nm and 450 nm, respectively, and performed in triplicate. The dashed line shows the half-maximal effective concentration (EC_{50}).

Table S1. Information of the H3N8 viruses isolated in this study.

NO.	Viruses	Abbreviation	Collection date	Isolation locations	Species	Sample numbers
1	A/wild duck/Shandong/1765/2017(H3N8)	WD/1765/17	November 7, 2017	Yellow River delta	Wild duck	503
2	A/wild duck/Shandong/1766/2017(H3N8)	WD/1766/17	November 7, 2017	Yellow River delta	Wild duck	
3	A/wild duck/Shandong/1895/2017(H3N8)	WD/1895/17	November 7, 2017	Yellow River delta	Wild duck	
4	A/wild duck/Shandong/1923/2017(H3N8)	WD/1923/17	November 7, 2017	Yellow River delta	Wild duck	
5	A/wild duck/Shandong/2003/2017(H3N8)	WD/2003/17	November 7, 2017	Yellow River delta	Wild duck	
6	A/wild duck/Shandong/2021/2017(H3N8)	WD/2021/17	November 7, 2017	Yellow River delta	Wild duck	
7	A/wild duck/Shandong/6252/2019(H3N8)	WD/6252/19	October 8, 2019	Yellow River delta	Wild duck	1045
8	A/wild duck/Shandong/6268/2019(H3N8)	WD/6268/19	October 8, 2019	Yellow River delta	Wild duck	
9	A/wild duck/Shandong/6271/2019(H3N8)	WD/6271/19	October 8, 2019	Yellow River delta	Wild duck	
10	A/wild duck/Shandong/6275/2019(H3N8)	WD/6275/19	October 8, 2019	Yellow River delta	Wild duck	
11	A/wild duck/Shandong/6277/2019(H3N8)	WD/6277/19	October 8, 2019	Yellow River delta	Wild duck	
12	A/wild duck/Shandong/6279/2019(H3N8)	WD/6279/19	October 8, 2019	Yellow River delta	Wild duck	
13	A/wild duck/Shandong/6280/2019(H3N8)	WD/6280/19	October 8, 2019	Yellow River delta	Wild duck	
14	A/wild duck/Shandong/6284/2019(H3N8)	WD/6284/19	October 8, 2019	Yellow River delta	Wild duck	
15	A/wild duck/Shandong/6293/2019(H3N8)	WD/6293/19	October 8, 2019	Yellow River delta	Wild duck	
16	A/wild duck/Shandong/11221/2019(H3N8)	WD/11221/19	December 5, 2019	Yellow River delta	Wild duck	1178
17	A/wild duck/Shandong/11397/2019(H3N8)	WD/11397/19	December 5, 2019	Yellow River delta	Wild duck	
18	A/gull/Shandong/W1511/2021(H3N8)	GL/W1511/21	October 10, 2021	Yellow River delta	Gull	806
19	A/gull/Shandong/W1513/2021(H3N8)	GL/W1513/21	October 10, 2021	Yellow River delta	Gull	
20	A/gull/Shandong/W1518/2021(H3N8)	GL/W1518/21	October 10, 2021	Yellow River delta	Gull	
21	A/gull/Shandong/W1519/2021(H3N8)	GL/W1519/21	October 10, 2021	Yellow River delta	Gull	

Table S2. Molecular characteristics of the H3N8 viruses in this study

	**HA (H3 Numbering)**			PB2		PB1		PA	NP	M1		NS1
	**Cleavage site**	I155T	T160A	E627K/V	D701N	R207K	H436Y	S/A515T	V41I	N30D	T215A	V149A
**WD/W1765/17**	PEKQTR/GLF	T	A	E	D	K	Y	T	I	D	A	A
**WD/W1766/17**	PEKQTR/GLF	T	A	E	D	K	Y	S	I	D	A	A
**WD/W1895/17**	PEKQTR/GLF	T	A	E	D	K	Y	T	I	D	A	A
**WD/W1923/17**	PEKQTR/GLF	T	A	E	D	K	Y	T	I	D	A	A
**WD/W2003/17**	PEKQTR/GLF	T	A	E	D	K	Y	T	I	D	A	A
**WD/W2021/17**	PEKQTR/GLF	T	A	E	D	K	Y	T	I	D	A	A
**WD/W6252/19**	PEKQTR/GLF	T	A	E	D	K	Y	T	I	D	A	A
**WD/W6268/19**	PEKQTR/GLF	T	A	E	D	K	Y	T	I	D	A	A
**WD/W6271/19**	PEKQTR/GLF	T	A	E	D	K	Y	T	I	D	A	A
**WD/W6275/19**	PEKQTR/GLF	T	A	E	D	K	Y	T	I	D	A	A
**WD/W6277/19**	PEKQTR/GLF	T	A	E	D	K	Y	T	I	D	A	A
**WD/W6279/19**	PEKQTR/GLF	T	A	E	D	K	Y	T	I	D	A	A
**WD/W6280/19**	PEKQTR/GLF	T	A	E	D	K	Y	T	I	D	A	A
**WD/W6284/19**	PEKQTR/GLF	T	A	E	D	K	Y	T	I	D	A	A
**WD/W6293/19**	PEKQTR/GLF	T	A	E	D	K	Y	T	I	D	A	A
**WD/W11221/19**	PEKQTR/GLF	T	A	E	D	K	Y	T	I	D	A	A
**WD/W11397/19**	PEKQTR/GLF	T	A	E	D	K	Y	T	I	D	A	A
**GL/W1511/21**	PEKQTR/GLF	T	A	E	D	K	Y	T	I	D	A	A
**GL/W1513/21**	PEKQTR/GLF	T	A	E	D	K	Y	T	I	D	A	A
**GL/W1518/21**	PEKQTR/GLF	T	A	E	D	K	Y	T	I	D	A	A
**GL/W1519/21**	PEKQTR/GLF	T	A	E	D	K	Y	T	I	D	A	A

Table S3. The HI antibody of the chickens and pigeons prior of infection studies.

Animal NO.	HI antibody titer of animal serum (log $_2$)							
	Chicken				Pigeon			
	H5(Re-13)	H5(Re-14)	H7(Re-4)	H9	H5(Re-13)	H5(Re-14)	H7(Re-4)	H9
#1	5	5	4	11	0	0	0	1
#2	4	3	5	11	0	0	0	1
#3	4	4	5	11	0	0	0	1
#4	3	4	4	11	0	0	0	1
#5	3	3	4	11	0	0	0	1
#6	2	2	3	11	0	0	0	0
#7	3	4	4	9	0	0	0	1
#8	2	3	5	11	0	0	0	0
#9	0	3	4	10	0	0	0	1
#10	3	5	6	10	0	0	0	1
#11	2	2	2	10	0	0	0	3
#12	0	4	5	11	0	0	0	1
#13	4	4	4	11	0	0	0	1
#14	3	3	3	11	0	0	0	1
#15	6	4	3	10	0	0	0	1
#16	2	4	5	11	0	0	0	1
#17	4	5	4	11	0	0	0	1
#18	2	3	4	11	0	0	0	0
#19	3	4	3	11	0	0	0	1
#20	2	5	5	11	0	0	0	1
#21	4	6	5	11	0	0	0	0
#22	2	5	4	11	0	0	0	3
#23	2	4	5	11	0	0	0	1
#24	2	4	5	11	0	0	0	3
#25	4	3	4	9	0	0	0	0
#26	1	2	3	11	0	0	0	2
#27	0	1	4	10	0	0	0	1
#28	2	3	4	11	0	0	0	0
#29	3	3	4	11	0	0	0	0
#30	2	3	3	10	0	0	0	1
#31	3	3	4	9	0	0	0	0
#32	2	2	4	10	0	0	0	0
#33	4	4	5	10	0	0	0	3
#34	3	4	5	11	0	0	0	1
#35	3	5	5	11	0	0	0	1
#36	4	5	6	11	0	0	0	1
#37	1	2	3	9	0	0	0	2
#38	4	4	5	9	0	0	0	0
#39	2	2	4	10	0	0	0	0

Table S4. Seroconversion of the chickens and pigeons inoculated or directed contacted with H3N8 viruses

Virus	Chicken seroconversion: positive/ total [a] (HI antibody titer)						Pigeon seroconversion: positive/ total [a] (HI antibody titer)					
	Inoculated group (d.p.i [b])			Contacted group (d.p.c[c])			Inoculated group (d.p.i)			Contacted group (d.p.c)		
	10	15	21	10	15	21	10	15	21	10	15	21
WD/W1895/ 17	1/5 (4, - [d], -, -, -)	2/5 (8, 32, -, -, -)	2/5 (2, 16, - , -, -)	0/5 (-, -, -, -, -)	1/5 (2, -, -, -, -)	0/5 (-, -, -, -, -)	0/5 (-, -, -, -, -)	0/5 (-, -, -, -, -)	0/5 (-, -, -, -, -)	0/5 (-, -, -, -, -)	0/5 (-, -, -, -, -)	0/5 (-, -, -, -, -)
WD/W6275/ 19	2/5 (32, 128, -, -, -)	3/5 (2, 16, 32, -, -)	3/5 (2, 4, 16, -, -)	0/5 (-, -, -, -, -)	0/5 (-, -, -, -, -)	0/5 (-, -, -, -, -)	0/5 (-, -, -, -, -)	0/5 (-, -, -, -, -)	0/5 (-, -, -, -, -)	0/5 (-, -, -, -, -)	0/5 (-, -, -, -, -)	0/5 (-, -, -, -, -)
GL/W1518/21	2/5 (2, 32, -, -, -)	3/5 (8, 16, 16, -, -)	2/5 (8, 16, -, -, -)	0/5 (-, -, -, -, -)	1/5 (8, -, -, -, -)	1/5 (4, -, -, -, -)	0/5 (-, -, -, -, -)	0/5 (-, -, -, -, -)	0/5 (-, -, -, -, -)	0/5 (-, -, -, -, -)	0/5 (-, -, -, -, -)	0/5 (-, -, -, -, -)

[a] The chicken or pigeon serum was collected on 10, 15, 21 dpi. The HI antibody titers were detected by HI assay. The tested H3N8 viruses were diluted to 4 HAU and then reacted with the chicken or pigeon serum, e.g., the WD/W1895/ 17 was diluted to 4 HAU and then reacted with the chicken serum of the WD/W1895/ 17 group.

[b] days post-inoculation.

[c] days post-contact.

[d] The HI antibody titer was negative.
